# Plasticity within the barrel domain of BamA mediates a hybrid-barrel mechanism by BAM

**DOI:** 10.1038/s41467-021-27449-4

**Published:** 2021-12-08

**Authors:** Runrun Wu, Jeremy W. Bakelar, Karl Lundquist, Zijian Zhang, Katie M. Kuo, David Ryoo, Yui Tik Pang, Chen Sun, Tommi White, Thomas Klose, Wen Jiang, James C. Gumbart, Nicholas Noinaj

**Affiliations:** 1 Interdisciplinary Life Science - PULSe, Purdue University, West Lafayette, IN 47907 USA; 2 Markey Center for Structural Biology, Department of Biological Sciences, Purdue University, West Lafayette, IN 47907 USA; 3 School of Physics, Georgia Institute of Technology, Atlanta, GA 30332 USA; 4 School of Chemistry and Biochemistry, Georgia Institute of Technology, Atlanta, GA 30332 USA; 5 Interdisciplinary Bioengineering Graduate Program, Georgia Institute of Technology, Atlanta, GA 30332 USA; 6 Electron Microscopy Core Research Facility, W125 Vet Med Building, University of Missouri, Columbia, MO 65211 USA; 7 Purdue CryoEM Facility, Suite 171, Hockmeyer Hall for Structural Biology, Purdue University, West Lafayette, IN 47907 USA; 8 Purdue Institute of Inflammation, Immunology and Infectious Disease, Purdue University, West Lafayette, IN 47907 USA; 9 Present Address: Dixie State University, 225 South 700 East, St. George, UT 84770 USA; 10 Present Address: Purdue University, 240S. Martin Jischke Drive, Hockmeyer Hall of Structural Biology, West Lafayette, IN 47907 USA

**Keywords:** Protein folding, Cryoelectron microscopy, Membrane proteins

## Abstract

In Gram-negative bacteria, the biogenesis of β-barrel outer membrane proteins is mediated by the β-barrel assembly machinery (BAM). The mechanism employed by BAM is complex and so far- incompletely understood. Here, we report the structures of BAM in nanodiscs, prepared using polar lipids and native membranes, where we observe an outward-open state. Mutations in the barrel domain of BamA reveal that plasticity in BAM is essential, particularly along the lateral seam of the barrel domain, which is further supported by molecular dynamics simulations that show conformational dynamics in BAM are modulated by the accessory proteins. We also report the structure of BAM in complex with EspP, which reveals an early folding intermediate where EspP threads from the underside of BAM and incorporates into the barrel domain of BamA, supporting a hybrid-barrel budding mechanism in which the substrate is folded into the membrane sequentially rather than as a single unit.

## Abstract (web summary)

The β-barrel assembly machinery (BAM) assists the folding and membrane insertion of bacterial outer membrane proteins. Here, the authors report structural characterization of BAM in lipid environment and in complex with the client protein EspP integrated into the barrel of BamA, providing insight into BAM mechanism of function.

## Introduction

**Fig. 1:**
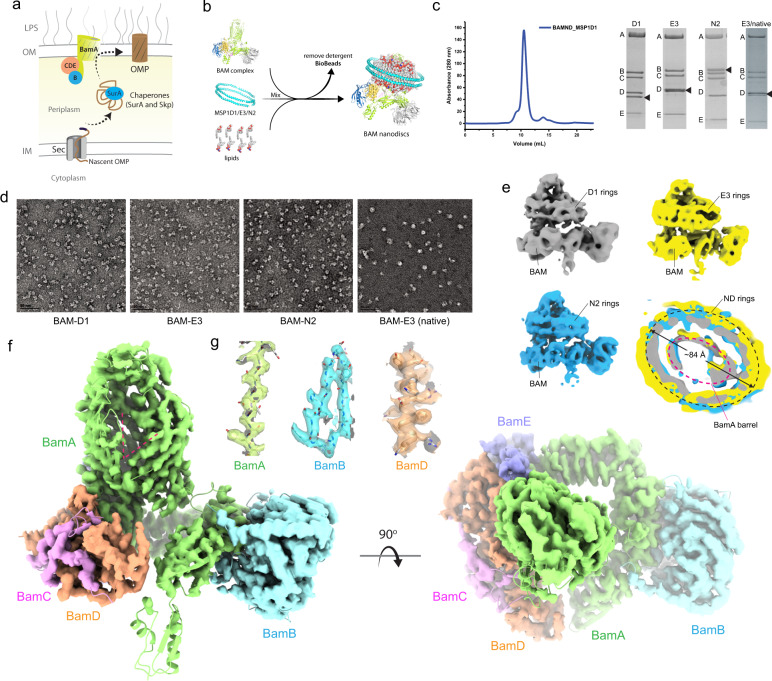
CryoEM structure of *E. coli* BAM in nanodiscs. **a** The role of BAM in the biogenesis of β-barrel outer membrane proteins. This figure is an adaptation from Imai et al.^48^ and is used under a CC BY 4.0 license; the image was modified to demonstrate the general pathway for BAM-mediated OMP biogenesis. **b** The procedure for forming BAM-inserted nanodiscs used for our EM studies. Source data are provided as a Source Data file. **c** A representative SEC trace from the purification of BAM-inserted nanodiscs, along with SDS-PAGE gels for each of the purifications from our study. The black triangles indicate the nanodisc proteins. **d** Negative-stain images for all BAM-inserted nanodisc samples in our study. The scale bars represent 50 nm. **e** CryoEM reconstructions of BAM-inserted D1, E3, and N2 nanodiscs, including a top-down cutaway view of a superposition of the three structures showing nearly identical densities for each of the different nanodiscs used. The red dashed line indicates the location of the barrel domain of BamA, while the black dashed line indicates the perimeter of the nanodisc density. **f** A refined model of BAM (PDB ID 7RI5) docked within the cryoEM 3D reconstruction of BAM in E3 nanodiscs at 4 Å resolution showing an outward-open conformation (red dashed lines), with an orthogonal view from the top of the barrel of BamA. **g** Representative electron density for BamA (residues 600–620), BamB (residues 58–77), and BamD (residues 164–181).

The outer membrane (OM) of Gram-negative bacteria is essential and its unique composition provides a protective barrier from the environment. In *E. coli*, the OM is asymmetric, composed of phospholipids in the inner leaflet and lipopolysaccharide (LPS) in the outer leaflet. The OM is composed almost exclusively of β-barrel outer membrane proteins (OMPs)^1–3^. Single OMPs vary in size from 8 to 36 strands and serve many roles within the cell including nutrient transport, signaling, and as virulence factors in pathogenic strains^2,4–10^. The biogenesis of these OMPs is mediated by the β-barrel assembly machinery (BAM), which has five-components termed BamA-E, with BamA being the central membrane-embedded component^11–14^ (Fig. 1a). The composition of BAM varies by species with only BamA, an OMP itself, being fully conserved across all Gram-negative bacteria^15,16^. BamA is thought to be the workhorse of the complex, however, it is still not known exactly what role the other components play^17,18^. Orthologs of BamA can be found in eukaryotic organelles such as mitochondria (Sam50) and chloroplasts (Toc75/Oep80) where they are believed to perform a related function^2,5,15,19–25^.

Structures of BAM solved by X-ray crystallography and cryo-electron microscopy (cryoEM) show that BamB-E interact with BamA along its periplasmic POTRA domains and that the complex is overall conformationally dynamic^26–29^. The structures of BamA revealed a lateral opening along the barrel seam (strands β1 and β16), which supported an earlier hypothesis that the barrel domain is directly involved in mediating OMP biogenesis, possibly through a strand templating mechanism^18,30,31^. The full extent of this lateral opening was observed in the structures of fully assembled BAM where the barrel domain of BamA was observed in both inward-open and outward-open states^17,26–29^. The exact role of these different states is still unknown, but cycling between them may drive either partially spontaneous or systematic insertion of substrate OMPs during biogenesis.

Given its role as a virulence factor in enterohemorrhagic *E. coli* O157:H7, the serine protease EspP has been studied extensively structurally and functionally^32–37^. EspP is a 12-stranded OMP belonging to the type Va secretion system subclass of autotransporters^38,39^. Like other classical autotransporters, EspP is composed of an N-terminal passenger domain and a C-terminal β-barrel domain. The passenger domain must be transported from the periplasm across the outer membrane to the outside of the cell, which is proposed to involve traveling through the barrel domain^40^. Studies have shown that BAM is required for the biogenesis of EspP by directly folding the barrel domain and assisting in the translocation of the passenger domain across the outer membrane^35,36,41^. Once translocated, the passenger domain is then released from the surface by cleavage of the polypeptide within the barrel domain of EspP^38,40^. Recent crosslinking studies have shown that the biogenesis of EspP by BAM involves direct physical contact with exposed strands of BamA at its lateral seam^41^. A similar observation has also been made for the biogenesis of Por1 by the conserved SAM complex in mitochondria^24^. These studies support the hypothesis that the biogenesis of new OMPs by BAM and SAM involves strand templating and β-strand augmentation, however, the exact details remain unresolved.

Two leading general mechanisms for how BAM performs its role in OMP biogenesis have been postulated^12,17^. In the first, BAM primarily acts as a catalyst on the membrane to mediate trafficking to the membrane and spontaneous insertion of unfolded or partially folded substrates. In the second, BAM still acts as a catalyst on the membrane, however, the BamA barrel also serves as a template for the systematic assembly of OMPs during folding and insertion into the membrane. The folding process is thought to be initiated when the β-signal (typically the last strand of the barrel domain) of the nascent OMP pairs with the exposed edge of the first strand of BamA following lateral opening^17^. Systematic assembly has been postulated to involve strand templating via β-augmentation to grow the substrate OMP barrel from that of BamA until biogenesis is terminated and the new OMP diffuses into the membrane^42^.

In this work, to resolve the mechanism BAM uses for OMP biogenesis, we present the structure of BAM in nanodiscs, with one structure being resolved in nanodiscs prepared from native membranes, for which we observe an outward-open conformation. Mutagenesis and crosslinking experiments demonstrate that plasticity of the barrel domain of BamA is essential for function, which is further supported by molecular dynamics (MD) simulations. We also report the structure of a BAM/EspP intermediate at the early stages of biogenesis, demonstrating integration of EspP into the barrel of BamA and further supporting a hybrid-barrel budding mechanism.

## Results

### Structures of BAM in nanodiscs

Until recently^43,44^, structures of BamA and of BAM had only been solved in detergents, which has yielded various conformational states^12,17^. Exactly which of these states is important for BAM function had been unclear as is what role they may play. To provide more insight into the state of BAM within a membrane bilayer, we prepared BAM-containing nanodiscs using *E. coli* polar lipids (Fig. 1b, c) and initially characterized the samples using negative-stain EM, then moved to cryoEM. Starting with MSP1D1 nanodiscs (D1, 11 nm) (Fig. 1d and Supplementary Fig. 1), we found very little space between BAM and the MSP1D1 belt. This was worrisome since the BAM-inserted nanodiscs are used for in vitro activity assays by several groups, however, the reconstruction suggested that there was not enough room for an OMP within the nanodisc. Therefore, we did a further analysis using cryoEM to compare the three different sizes of BAM-inserted nanodiscs including the MSP1D1 (D1; 11 nm), MSP1E3D1 (E3; 13 nm), and the MSP2N2 (N2; 17 nm) (Fig. 1e and Supplementary Fig. 2). Our initial structures ranged in resolution from 6.9 to 8.0 Å (Supplementary Table 1) with minimal conformational changes, as demonstrated by RMSD measurements of the refined structures ranging from 1.5 to 2.2 Å. Based on the best resolution in our initial studies, we further optimized the MSP1E3D1 sample and improved the resolution of the reconstruction to 4 Å, which enabled us to observe individual strands of the barrel domain of BamA and side-chain density for most residues of BAM (RMSD of 2.4 Å with PDB ID 5LJO) (Fig. 1f, g, and Supplementary Fig. 3). Surprisingly, despite using significantly different sizes of nanodiscs, we observed almost no space between the barrel domain of BamA (~5 nm in diameter along the longest axis) and the nanodisc scaffold proteins (ranging from 11 to 17 nm in diameter) (Fig. 1e, bottom-right panel). However, we rationalized that our observations were exactly what we should expect given that most of the nanodisc density is likely being averaged out during the reconstructions. To further support this notion, we performed computational simulations showing that if BAM is randomly positioned within circular nanodiscs and then aligned along BAM itself, the majority of the nanodisc density is indeed averaged out, leaving density with the strongest intensity observable only along the perimeter of the barrel domain of BamA (Supplementary Fig. 4). This idea would presumably apply to other proteins that are randomly positioned within nanodiscs. To further probe the structure of BAM in a more native-like environment, we also determined the structure of BAM in MSP nanodiscs prepared directly from *E. coli* cellular membranes (Fig. 1d and Supplementary Figs. 2 and 5). Importantly, we observed an outward-open conformation in all reconstructions, indicating that the membrane-inserted state of BAM is indeed the outward-open state.

### Dynamics of BAM from MD simulations

Comparison of our cryoEM structures to those reported previously in detergent^26–29^ shows that only the outward-open conformation is observed within the lipid bilayer, however, with some minor movements along the periplasmic portions relative to the barrel of BamA (Supplementary Fig. 2). To probe the dynamics of BAM here and to gain insight into the role of the accessory proteins BamB-E, we performed molecular dynamics (MD) simulations for BAM complex systems in a native membrane environment starting in either the outward-open state, denoted BAM(out), or the inward-open state, denoted BAM(in), using existing high-resolution structures (PDBs 5LJO^28^ and 5D0O^27^, respectively). In addition to full BAM, we deleted either BamB, BamC, BamD, or BamE for systems starting in the inward-open state and BamB for a system starting in the outward-open state; each was simulated for 6 μs × 3 replicas (126 μs in total). While only three simulations provide insufficient statistics to reliably quantify the probabilities of observed events, they can at least demonstrate their possibility. Furthermore, comparisons can be made between systems when observations are broadly consistent between replicas.

**Fig. 2:**
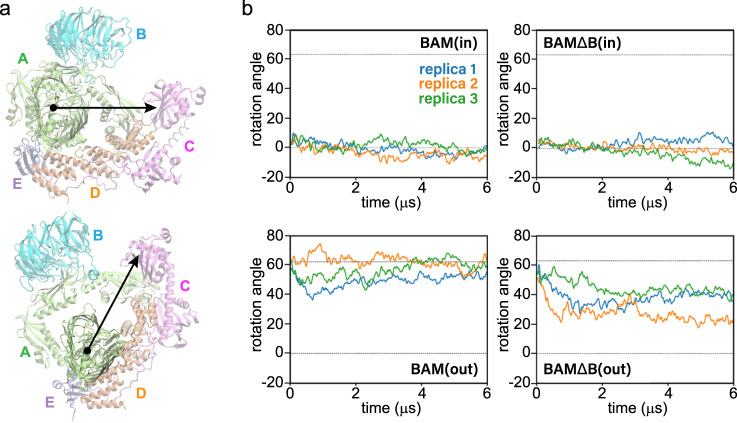
Rotational dynamics of BAM from MD simulations. **a** Rotational dynamics of BAM in the membrane plane showing the subunit positions overlaid onto an extracellular view of the respective structures (top, inward-open; bottom, outward-open). **b** Rotation angle of the periplasmic domain ring in the membrane plane calculated using the average angle made by BamB (when present), BamC, BamD, and BamE with respect to BamA. The angle of these lipoproteins in the structure 5D0O is used as a reference and is measured counterclockwise as viewed from the extracellular space. The dotted lines are the rotation angles in the structures used to initialize the inward-open state (0°; PDB 5D0O) and the outward-open state (63°; PDB 5LJO). Plots are from three independent 6-μs simulations demonstrating a range of possible dynamics on this time scale.

**Fig. 3:**
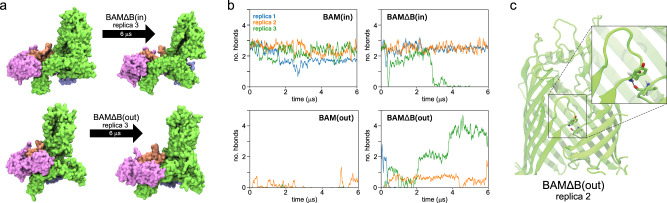
Lateral seam dynamics of the BAM from MD simulations. **a** Snapshots showing notable conformational state transitions observed in MD simulations. **b** Moving average of the hydrogen bonds between the backbones of BamA lateral seam strands. Plots are from three independent 6-μs simulations demonstrating a range of possible dynamics. **c** Snapshot of the BamA β-barrel from the second replica of BAMΔΒ(out) highlighting a single backbone hydrogen bond formed between residues Y432 and I806.

The simulations revealed that the outward-open state is more dynamic than the inward-open state (Fig. 2), and that in the absence of BamB, the two states can partially inter-convert (Fig. 3). Accessory proteins of systems starting in the outward-open state are more dynamic, exploring a greater area in the membrane plane than those starting in the inward-open state (Supplementary Fig. 6). The difference in dynamics between conformational states can also be seen in the rotation angle of the accessory proteins (Fig. 2a and Supplementary Fig. 7). These proteins, along with BamA’s five POTRA domains, form a “periplasmic ring”, which is rotated about 60° counterclockwise (viewed from the extracellular side) in the outward-open state compared to the inward-open state (Fig. 2b)^27^. In simulations, the inward-open states stay near or below 0° (the initial position). In particular, the rotation angle of one of the BAMΔD(in) replicas decreases by over 20°, which may limit its ability to transition to an inward-open state (Supplementary Fig. 7). BamD is known to be essential^45^, and substrate binding to BamD alters BamA’s conformation^46^. In contrast to most inward-open states, the outward-open states fluctuate considerably. Despite these fluctuations, all BAM(out) replicas end within 5° of their starting state; in contrast, the rotation angles for the BAMΔΒ(out) replicas decrease by 25–35°, i.e., halfway to the inward-open state. Further examination of two of these replicas reveals partial conformational switching in which the BamA β-barrel lateral seam closes. While in one of these replicas, significant contact is made at the seam (Fig. 3a and Supplementary Fig. 8), in the other, extracellular loop 1 of BamA makes only tenuous contact with β16, reminiscent of the closed state in recently reported structures of Sam50^25^ (Fig. 3c).

Although no large changes in rotation angle were observed for any BAMΔΒ(in) replicas, the lateral seam in one replica opened. However, rather than adopting an outward-open-like sheared conformation, the strands separate in a parallel fashion reminiscent of previously reported open states from MD simulations of BamA alone^18,30^ (Fig. 3a). The conformational transitions observed can be further quantified by tracking the hydrogen bonds at the BamA β-barrel seam over time (Fig. 3b and Supplementary Fig. 9). Replica 3 of BAMΔΒ(in) loses the hydrogen bonds at the β-barrel-seam around 3 μs into the simulation, and the resultant opening persists for the remainder of the simulation. The β-barrel seam in replica 3 of BAMΔΒ(out) closes over time, forming two hydrogen bonds at the β-barrel seam at ~2 μs, which rises to four hydrogen bonds at 4 μs that are then maintained. Replica 2 of BAMΔΒ(out), on the other hand, maintains an average of less than one hydrogen bond, exemplifying the tenuous nature of its closed state shown in Fig. 3c.

We also tracked the position of BamA’s POTRA5, which is immediately below the β-barrel. While the positions of POTRA5 in simulations of BAM(in), BAM(out), and BAMΔΒ(in) are relatively static, that of BAMΔΒ(out) explores a much larger range, including overlapping with those of BAM(in) and BAMΔΒ(in) (Supplementary Fig. 10a). When looking at simulations individually, the greatest movement of POTRA5 is seen for the two replicas of BAMΔΒ(out) for which the β-barrel seam closes (Supplementary Fig. 10b). In contrast, relatively little movement of POTRA5 is seen for the replica of BAMΔΒ(in) in which the β-barrel seam opens, as also recently seen for Sam50^25^. Thus, the connection between the β-barrel seam and movement of the POTRA5 domain, if any, remains unclear.

### Probing conformational plasticity of BamA

**Fig. 4:**
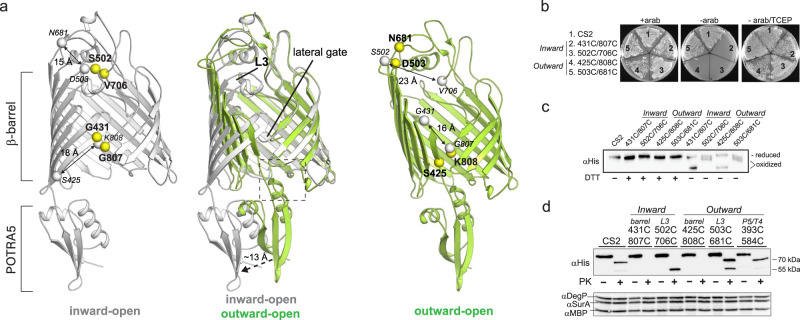
Probing conformational plasticity of the BamA barrel domain. **a** A superposition of the inward-open and outward-open conformations of the barrel domain of BamA (middle). The left and right panels indicate the locations of crosslink mutations made in these studies, with the crosslinks designed to lock each specific conformation shown in yellow. The gray spheres indicate the locations of the crosslink mutant pairs in the opposite conformation, which are separated by 15–23 Å. **b** Plate assays using JCM166 cells transformed and plated onto LB agar plates with the C2S control and crosslink mutants locking each of the conformations designed in panel **a**. Each was performed at least in triplicate with representative images shown. **c** Analysis of the crosslink mutants by western blot on a 5% SDS-PAGE gel showing the direct observation of the crosslinked species (oxidized), which can be reduced in the presence of DTT. Assays were performed at least in triplicate with a representative image shown. Source data are provided as a Source Data file. **d** Surface shaving experiments with proteinase K (PK), followed by western blot analysis, verified each of the indicated mutants are surface exposed with no disruption in biogenesis. Assays were performed at least in triplicate with a representative image shown. Source data are provided as a Source Data file.

To determine the importance of the conformational states for function, we used mutagenesis and crosslinking studies to target two regions of BAM: the barrel of BamA, and the position of POTRA5 (Fig. 4a, left panel). Here, we utilized complementation assays using JCM166 cells, which have endogenous BamA is under an arabinose promoter, to analyze phenotypes of cells harboring plasmids encoding BamA mutants^11,18^. Guided by the BAM structures found in the two conformational states, we first probed the inward-open state by preparing paired disulfide mutants containing S502C/V706C (loops 3 and 6) and G431C/G807C (lateral seam) (Fig. 4a, left panel). Neither mutant was able to grow in the absence of arabinose, however, both could be rescued with TCEP and could be directly observed by SDS-PAGE, indicating that the crosslink mutants were indeed forming in vivo and preventing BAM function (Fig. 4b, c). Next, we probed the outward-open state by preparing disulfide mutants containing D503C/N681C (loops 3 and 6) and S425C/K808C (lateral seam) (Fig. 4a; right panel). Despite directly observing the crosslinks by SDS-PAGE confirming they were forming in vivo, only the S425C/K808C crosslink at the lateral seam abolished BAM function, while the D503C/N681C crosslink grew similar to the cysteine-less C2S control even in the absence of arabinose (Fig. 4b, c and Supplementary Fig. 11). Additionally, surface shaving experiments were performed on these sets of crosslink mutants to confirm they were surface exposed, and that the biogenesis of each mutant was not disrupted as monitored by DegP and SurA levels which remained unchanged. This set of experiments revealed that our L3 inward-open mutant exhibited a differing digest signature after proteinase K (PK) treatment than the C2S control, with the L3 outward-open mutant exhibiting a mix of the two, but predominantly C2S (Fig. 4d). To further explore our observations for the D503C/N681C crosslink, MD simulations revealed that despite the formation of this crosslink, it was likely insufficient to fully lock the barrel in the outward-open state. Instead, the barrel domain maintained sufficient plasticity to accommodate both states and importantly, did not disrupt cycling at the lateral seam (Supplementary Fig. 12), agreeing well with the results from the proteinase K digest assays.

**Fig. 5:**
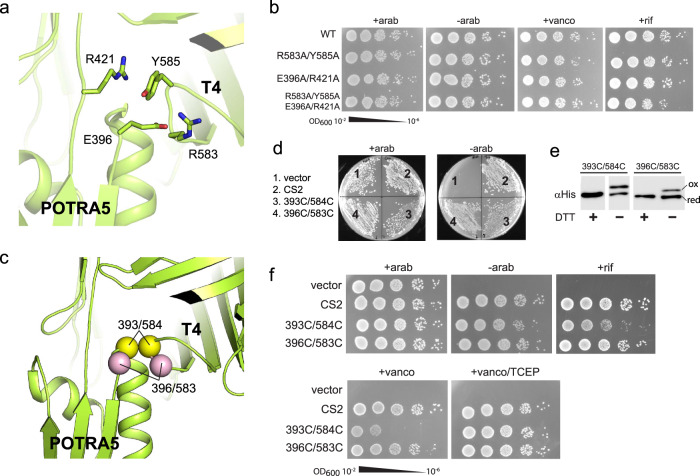
Probing the interactions between BamA POTRA5 and turn 4. **a** Zoomed view of the interaction between POTRA5 and turn 4 (T4) in the outward open state (dashed box from Fig. 3a, left panel), with four putative ‘lock’ residues shown in stick. **b** JCM166 cells were transformed with each of the indicated plasmids and serially diluted and spotted onto LB-agar plates with the indicated conditions. Each was performed at least in triplicate with representative images shown. **c** Zoomed view of the interaction between POTRA5 and turn 4 (T4), with the location of the two crosslink pairs indicated by yellow (393/584) and pink (396/583) spheres. **d** Plate assay using JCM166 cells transformed with each crosslink mutant and plated onto LB agar plates with and without arabinose. Each was performed at least in triplicate with representative images shown. **e** Analysis of the crosslink mutants by western blot on a 5% SDS-PAGE gel showing the direct observation of the crosslinked species (ox), which can be reduced (red) in the presence of DTT. Assays were performed at least in triplicate with a representative image shown. Source data are provided as a Source Data file. **f** JCM166 cells were transformed with each of the crosslink mutants and serially diluted and spotted onto LB-agar plates with the indicated conditions. The crosslink mutants were challenged with the antibiotics vancomycin (vanco) and rifampicin (rif) to identify any subtle changes in the outer membrane integrity. Each was performed at least in triplicate with representative images shown.

In the BAM structures reported to date, POTRA5 of BamA is observed in two positions that are ~13 Å apart: (i) away from the barrel domain (inward-open) and (ii) tucked up under the barrel domain making contact with turn 4 (T4; outward-open), which was thought to assist in shifting the barrel domain from inward-open to outward-open (Fig. 4a (left panel) and 5a). To probe the importance of the latter position, we mutated four ‘lock’ residues to alanine along POTRA5 and T4 that were well positioned to form strong interactions which included (i) a salt bridge formed between E396 (POTRA5) and R583 (T4) and (ii) a cation-pi stacking interaction between R421 (POTRA5) and Y585 (T4) (Fig. 5a). Surprisingly, neither single-, double-, or quadruple-alanine mutants for POTRA5/T4 had an observable phenotype in our plate assays. Therefore, we moved to serial dilutions and challenged the mutants with both vancomycin and rifampicin to investigate if these mutants may have membrane defects caused by a disruption in BamA function or biogenesis (Fig. 5b). But again, no effect was observed, suggesting that the POTRA5/T4 interaction is not essential for BAM function. However, our results seemingly contradict reported studies on BAM that indicated that crosslinking POTRA5 to T4 abolished BAM function, which led to the notion that cycling of POTRA5 between the two observed positions was required for function^27^. To probe this further, using the BAM structures as guides, we created two different disulfide crosslinks between POTRA5 and T4 consisting of E396C/R583C and the previously reported G393C/G584C (Fig. 5c). Contrary to previous studies, the results of our plate assays show that both crosslink mutants are able to grow even in the absence of arabinose (Fig. 5d). Furthermore, the crosslink species can be directly observed by SDS-PAGE/Western blot analysis, indicating that the crosslink mutants were indeed forming in vivo and surface exposed, yet were unable to disrupt BAM function (Figs. 4d and 5e). To observe if subtle changes in the membrane integrity may explain the discrepancies compared to previous reports, we performed serial dilutions of the crosslink mutants and challenged them with vancomycin and rifampicin (Fig. 5f). While again no effect was observed with the E396C/R583C mutant, we did observe susceptibility of the G393C/G584C mutant, particularly with vancomycin, suggesting that this mutant may be allowing leakiness of the membrane, however, not directly preventing BAM function. Additionally, a simulation of the G393C/G584C crosslink mutant reveals that despite this constraint, the barrel domain of BamA is still able to cycle between the outward-open and inward-open states with only minor perturbations (Supplementary Fig. 12). Taken together with the cryoEM structures in nanodiscs, our studies show that contrary to previous reports, physical separation of POTRA5 from T4 is unlikely and not essential for BAM function. Furthermore, our studies support that the in vivo resting state of BAM is the outward-open state and that conformational plasticity of the barrel domain of BamA, particularly at the lateral seam, is essential for function.

### Structure of a BAM/EspP hybrid-barrel intermediate

**Fig. 6:**
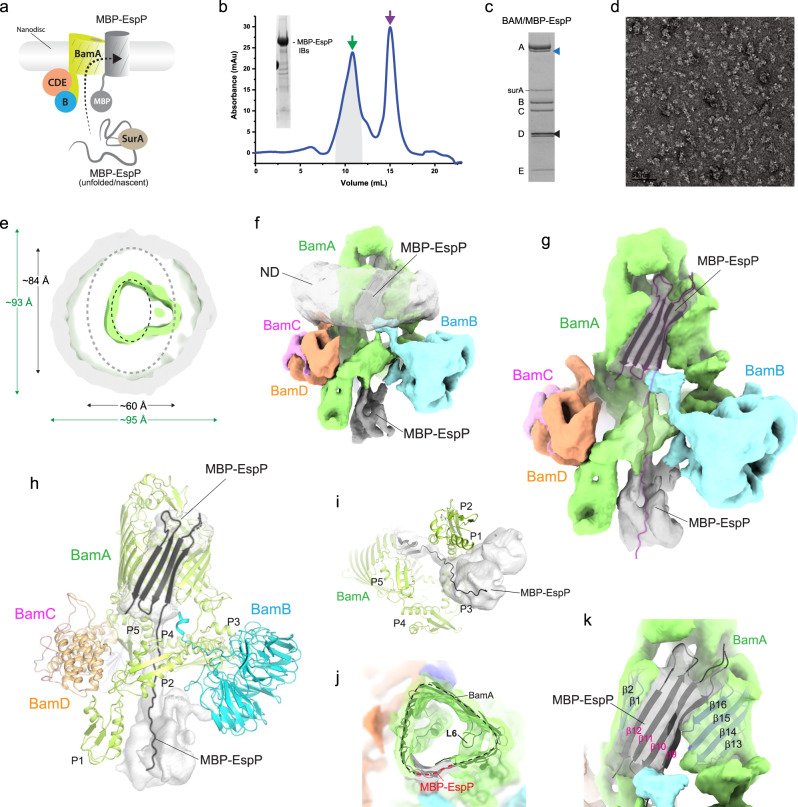
Low-resolution BAM/MBP-EspP structure from in vitro refolding assay. **a** Summary of the in vitro assay used for inserting MBP-EspP into BAM-nanodiscs. This figure is an adaptation from Imai et al.^48^ and is used under a CC BY 4.0 license; the image was modified to reflect the use of MBP-EspP in this set of experiments. **b** SEC trace of Bam/MBP-EspP purification after in vitro refolding. The green allow indicates the elution of the complex, while the purple arrow indicates the elution of excess surA. The inset shows SDS-PAGE analysis demonstrating the purity of the MBP-EspP inclusion bodies used for refolding. Source data are provided as a Source Data file. **c** SDS-PAGE gel of the MBP-EspP/BAM-inserted nanodiscs used for EM studies. The black triangle indicates the MSP protein while the blue triangle indicates MBP-EspP. Source data are provided as a Source Data file. **d** Negative-stain image of the MBP-EspP/BAM-inserted nanodiscs. The scale bar represents 50 nm. **e** A cutaway view of the 7.0 Å resolution BAM/MBP-EspP structure showing enlarged nanodisc density and triangular shape of the hybrid-barrel compared to the BAM-only structure. The position of the BamA barrel in the BAM-only structure is indicated by the black dashed oval; the position of the nanodisc density for the BAM-only structure is indicated by the gray oval. **f** The map from the cryoEM reconstruction to 7 Å resolution, with each component color coded. **g** The segmented map with the nanodisc density removed for clarity, depicting the extra density for the MBP-EspP substrate (gray map with ribbon in purple outline). **h** The nanodisc BAM/MBP-EspP intermediate complex highlighting only the density for the MBP-EspP component, modeled as four strands of the EspP barrel with the N-terminal region trailing down under BAM to a globular region of density. **i** A simplified view from under the hybrid-barrel. **j** Zoomed view of the triangular shape of BamA/EspP hybrid-barrel. **k** Zoomed view of the modeled four strands of EspP integrated into the barrel of BamA.

Recent in vivo studies have shown that a fusion construct of the barrel domain of EspP with an N-terminal maltose-binding protein (MBP) (MBP-EspP) could effectively be stalled on BAM during biogenesis, with the MBP tether preventing proper assembly and cleavage of the passenger domain helix^41^. To investigate the mechanism BAM uses for OMP biogenesis further, we used in vitro assays to fold the MBP-EspP substrate into BAM-inserted nanodiscs for structural analysis (Fig. 6a–c). These samples were first analyzed using negative-stain EM which showed well separated particles suitable for further EM analysis (Fig. 6d). CryoEM studies were then performed where we observed a mix of particles with and without MBP-EspP in our 3D sorting, with one 3D class having noticeably larger nanodisc density indicating the presence of the MBP-EspP substrate (Fig. 6e, f, and Supplementary Fig. 13). The final refined maps had a resolution of 7 Å, revealing not only larger nanodisc density, but the presence of a triangular shaped membrane domain, rather than the oval shape we have observed previously (Figs. 1e and 6e, j). The BAM structure was then refined against this density map which showed a widening of the barrel domain of BamA (Fig. 6f, g). Guided by the refined BAM structure and the available density remaining, we found we could accommodate at most four strands from EspP. Therefore, we modeled strands β9–12 of EspP pairing β12 (β-signal) with the first strand of BamA, much like what was observed with the recent BamA/BAM cryoEM structure^42^ (Fig. 6g, h, j, k). The C-terminal region of the barrel domain of BamA, which includes the conserved loop 6 (L6) and its interaction with the opposite wall, appears to swing out as a rigid body to accommodate the extra density within the barrel (Fig. 6j). It is not known exactly why we observed EspP stalled with only four strands, rather than with most of the barrel as in the original design^41^. However, one possible explanation is that the pressure of the surrounding lipids within the fixed-volume nanodisc created sufficient lateral force which limited further processing.

We also observed additional density in this reconstruction extending down from the modeled four EspP strands to underneath the barrel domain, indicating that the pathway by which the MBP-EspP substrate associates with BAM during biogenesis is from under the barrel domain of BamA (Fig. 6g, h and i). While a large globular-like density was observed which loosely resembled a partial MBP domain, we did not model MBP, rather only an elongated poly-alanine polypeptide extending from strand β9 of EspP. Given that this density was not observed in any of our other cryoEM structures, we postulate that this density reveals the pathway for substrate entry to BAM during folding.

**Fig. 7:**
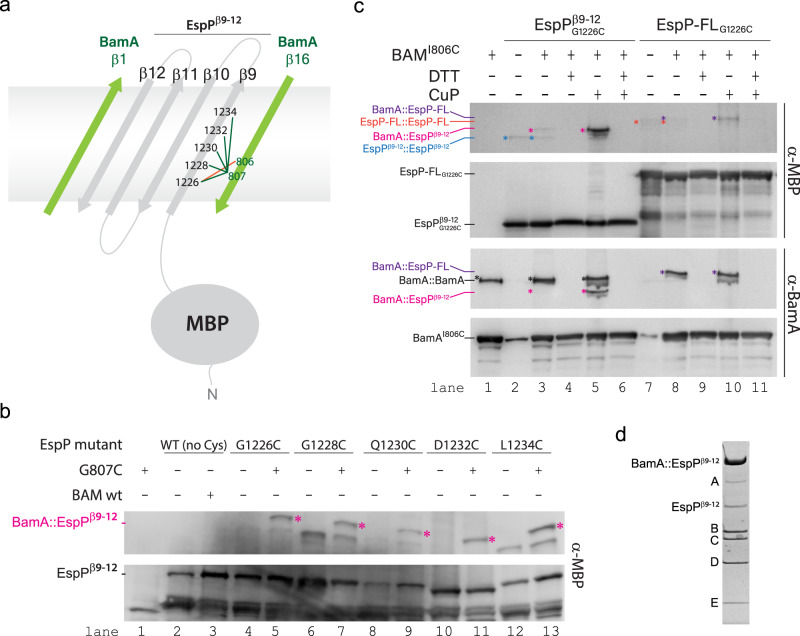
In vivo screening for the BAM/MBP-EspP^β9–12^ intermediate complex. **a** Schematic depicting the experimental design for the isolation of the BAM/MBP-EspP^β9–12^ intermediate complex. Crosslinking initially focused on residue 807 of β16 of BamA to residues 1226, 1228, 1230, 1232, and 1234 of β9 of EspP (green lines). The final crosslink leading to the high resolution structure was for residue 806 of BamA and residue 1226 of β9 of EspP (orange line). **b** Using a dual expression system, formation of the BAM/MBP-EspP^β9–12^ intermediate was monitored by SDS-PAGE and western blot analysis, with the crosslinked intermediate observed as a high molecular weight band (magenta). The crosslink at residue 1226 of EspP was purified for structure determination. Source data are provided as a Source Data file. **c** To optimize the stability of the crosslinked intermediate for structure determination, an additional crosslink was prepared on residue 806 of β16 of BamA with residue 1226 of β9 of EspP. SDS-PAGE and western blot analysis using α-MBP and α-BamA antibodies demonstrates that this crosslink forms in full length (FL)-EspP (lanes 8 and 10), yet more efficiently in the truncated construct (lanes 3–5). The crosslinks were enhanced by washing cells with copper o-phenanthroline (CuP) and reduced using DTT. Source data are provided as a Source Data file. **d** The BAM/MBP-EspP^β9–12^ crosslinked intermediate was purified by Ni-NTA and MBPTrap and verified by SDS-PAGE by the presence of the higher molecular weight BamA/EspP^β9–12^ crosslinked species. Source data are provided as a Source Data file.

**Fig. 8:**
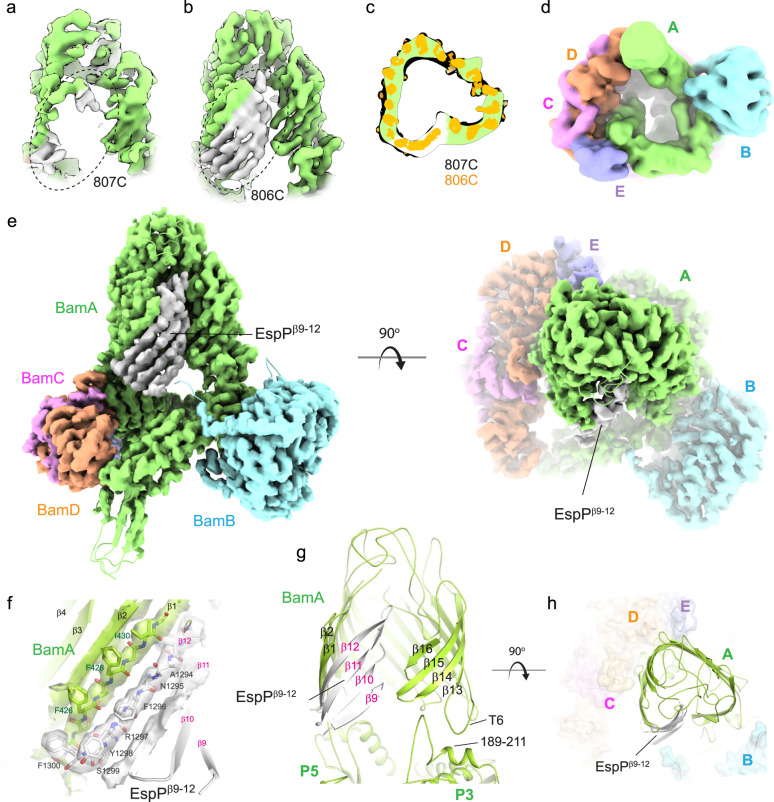
High-resolution structure of the BAM/MBP-EspP^β9–12^ hybrid-barrel early intermediate. CryoEM map along the lateral seam within the BAM/MBP-EspP^β9–12^ structure with crosslinking between **a** residue 807 of β16 of BamA (green) and residue 1226 of β9 of EspP (gray) and **b** residue 806 of β16 of BamA and residue 1226 of β9 of EspP. The dashed oval shows the increase in order observed with crosslinking at residue 806 of BamA. **c** A top-down cutaway view comparing the non-sharpened map from panel **a** (green is the BamA barrel; white is EspP^β9–12^) with the map in panel **b** (orange), depicting the same state observed for both crosslinking sites. **d** A bottom view of a down-filtered map (7 Å) from panel **b**; no extra density is observed as was seen for the structure from the in vitro assay (Fig. 6f–i). **e** Orthogonal views of the 3.4 Å resolution MBP-EspP^β9–12^ cryoEM map/structure showing the integration of EspP^β9–12^ within the expanded barrel of BamA, forming a hybrid-barrel early intermediate. The last strand of EspP^β9–12^ (β12) pairs with the first strand of BamA (β1). The N-terminal strands of EspP^β9–12^ then curve towards the inside of the barrel, which would assist in sealing the hybrid-barrel during biogenesis to maintain membrane integrity. **f** Zoomed view of the four strands of EspP^β9–12^ integrated into the barrel of BamA. The density for β1 of BamA and β12 of EspP^β9–12^ is shown as a transparent isosurface, allowing unambiguous assignment of the residues. **g** View of the BamA/EspP^β9–12^ barrel with strand arrangements. The interaction between turn 6 (T6) and residues 189-211 of POTRA5 (P3) is shown which may stabilize the expansion of the C-terminal region of the BamA barrel during OMP biogenesis. **h** Zoomed-in view of the triangular shape of BamA/EspP^β9–12^ hybrid-barrel, showing excellent agreement with the low-resolution structure from Fig. 6j.

Efforts to improve the resolution using our in vitro method were unsuccessful. Alternatively, we developed a dual expression system to purify a crosslinked BAM/MBP-EspP complex directly from bacterial membranes. We used our low resolution structure as a guide to design disulfide crosslinks between β16 of BamA and β9 of EspP using a construct of MBP-EspP containing only the last four strands of the barrel domain (EspP^β9–12^). We initially tested crosslinks between BamA β16 (G807) and EspP β9 at sites G1226, G1228, Q1230, D1232, and L1234 (Fig. 7a). Small-scale expression tests were performed, and SDS-PAGE of whole cell lysates followed by Western blot analysis showed that all of the sites produced a distinct crosslinked species for BamA/MBP-EspP (Fig. 7b, lanes 5, 7, 9, 11, 13) not present in the controls (Fig. 7b, lanes 1, 2, 3, 4, 6, 8, 10, 12). Large-scale expression was then performed in BL21(DE3) cells and the complexes purified using tandem Ni-NTA/MBPTrap columns. Only the crosslinked complex between BamA (G807C) and MBP-EspP^β9–12^ (G1226C) produced sufficient yields for structural studies. A cryoEM reconstruction of the complex was performed to 4.5 Å resolution, however, only sparse density was observed for EspP (Fig. 8a).

To improve the order of the EspP substrate, we then rationalized that moving the crosslink site to I806, which points into the membrane, could allow a more stable interaction (Fig. 7a). We showed using SDS-PAGE followed by western blot analysis using both α-BamA and α-MBP antibodies that the crosslink between BamA (I806C) and MBP-EspP^β9–12^ (G1226C) could also be readily formed (Fig. 7c, lane 3), which could be further enhanced in the presence of copper o-phenanthroline (Fig. 7c, lane 5). Similarly, the crosslink could be observed in a construct of the full length barrel domain of EspP (Fig. 7c, lanes 8, 10), confirming our crosslinked species represented a naturally occurring folding intermediate. Large-scale purification of this optimized complex was then performed (Fig. 7d), and grids prepared for cryoEM analysis. High-resolution data was collected on a Titan Krios equipped with an energy filter and K3 direct electron detector, consisting of 4405 micrographs with 956 K initial particles. Following iterative 2D and 3D classification, 149 K particles were used for the final reconstruction and refinement to 3.4 Å resolution (Fig. 8b, e and Supplementary Fig. 14).

Comparison of the complex structures using BamA crosslink site 807 versus 806 showed a significant improvement in the observable high resolution details for the strands of EspP in the 806 complex (Fig. 8a, b). Comparison of a low-pass filtered map of the 807 complex to the high resolution 806 structure verified that the overall conformations of the two structures are essentially the same (Fig. 8c), indicating that the change in crosslinking site served to stabilize the substrate, rather than trapping an alternate conformational state. In this improved structure, we could clearly observe strands β9–12 of EspP and unambiguously assign residues for β10-β12 (Fig. 8e, f). Loop 5 is mostly disordered, although appears to curl inward towards the lumen of the BamA barrel domain. This state represents what appears to be an early intermediate of the biogenesis of EspP by BAM and demonstrates the formation of a hybrid-barrel between EspP and BamA. The β-signal of EspP was found to be fully paired with the first strand of BamA, with the side chain of the terminal residue F1300 extending across and over G424 of strand β1 of the barrel domain of BamA, positioned in proximity behind F426 (Fig. 8f). No overhang was observed, as was seen in the recent structure of BAM in complex with a substrate BamA^42^. Whether or not this overhang may assist BAM in identifying BamA versus other substrates requires additional studies, however, these observations may offer some explanation for why a terminal phenyalanine is highly conserved in substrate OMPs in *E. coli* and Neisseria^47^. Additionally, we observed that the expanded barrel of BamA appears to be stabilized by interactions between turn 6 (T6) with POTRA3 (residues189–211) (Fig. 8g). While the triangular shape of the hybrid-barrel in this high resolution structure agrees remarkably well with the low-resolution reconstruction from the in vitro assay (Figs. 6j and 8h), we did not observe any additional density beneath the barrel domain of BamA at high resolution or after a 7 Å low-pass filter of the density map (Fig. 8d).

## Discussion

Given its essential role in the biogenesis of the outer membranes of Gram-negative bacteria, BAM is an exciting target for antibiotic discovery and development. Several recent studies have reported novel molecules targeting BAM, demonstrating the potential of future development, particularly against multidrug resistant strains of bacteria^48–50^. To support these therapeutic efforts and to elucidate the complex mechanism for how BAM performs its role in the cell, we report cryoEM structures of BAM in nanodiscs composed of polar lipids and native membranes, allowing us to conclusively identify the conformation of BAM within the bacteria membrane. Our results align with other recent reports showing BAM in an outward-open state^43,44^, which would fully expose the edge of β1 of BamA to promote direct interaction with the β-signal of incoming substrate OMPs to initiate biogenesis. However, these results seemingly conflict with the recently reported structure of BAM in saposin nanodiscs^44^, where an unusual conformation was observed that is essentially the outward-open state with the exception of strands β1–4 of the barrel domain of BamA having flipped to close the top of the barrel. Evidence that this is a minor modification of the outward-open state is supported by an RMSD of 1.6 Å of this structure with our high-resolution cryoEM structure, while an RMSD of 5.4 Å was calculated compared to the inward-open structure (PDB ID 5D0O). Whether the different properties of the saposin versus MSP1E3D1 nanodiscs allowed ordering of β1–4 of BamA remains to be determined (Supplementary Fig. 15). And contrary to the other recent cryoEM structures of BAM in MSP1D1 nanodiscs^43^, we did not observe an ensemble of clearly distinguishable conformations within the periplasmic domains of BAM. However, such motion is not surprising given that studies have already reported that the POTRA domains of BamA are dynamic^17,26,27,29,51–55^. In this same study, it was proposed that thinning of the membrane and distortion of the MSP belt was observed in these low resolution structures. Rather, the observations may align more with the averaging effect of the reconstructions we observed in our work here (Supplementary Fig. 4), although we do agree that BAM thins the membrane, as has been demonstrated previously^18,30,53,54,56,57^.

Further, we probe the conformational plasticity of BAM in vivo using mutagenesis and crosslinking studies, locking BAM in both outward-open and inward-open conformations, and demonstrating that contrary to previously reported studies, crosslinking POTRA5 to T4 does not disrupt function. This can be attributed to plasticity within the barrel domain of BamA, which is able to accommodate such restraints, as further demonstrated by our MD simulations studies. These studies show that there is a difference in the dynamics between the two main conformational states, with the outward-open state consistently exhibiting greater fluctuations than the inward-open state. We also observe that the accessory proteins, especially BamB, assist the complex in maintaining its conformational state, whether inward- or outward-open. In the absence of BamB, we observe partial conformational switching from inward- to outward-open, and vice versa in at least one of the replicas of each. Thus, we conclude that the BAM complex is more dynamic in the absence of BamB. Although two crystal structures were solved in the outward-open state in the absence of BamB, we note that both are stabilized by crystal contacts (Supplementary Fig. 16). This data matches well with the cryoEM structures presented here, where all structures of BAM within a membrane bilayer were found in the outward-open state. While we performed similar analyses for systems missing other accessory proteins including BAMΔC(in), BAMΔD(in), and BAMΔE(in) (Supplementary Figs. 6–10), removal of BamB had the most dramatic effect on dynamics. While BamB is not essential for viability, our results here support studies demonstrating that a lack of BamB in *E. coli* strongly impairs BAM function^11,58^, as well as reduces the rate of OMP insertion in proteoliposomes^59^ and greatly diminishes the assembly of BAM precincts^60^. Together, our work demonstrates the role of the accessory proteins in modulating the conformational state of BAM in *E. coli*, and highlight the importance of plasticity within the barrel domain of BamA for function, particularly along the lateral seam. This plasticity would facilitate BamA’s ability to structurally adapt to the budding OMPs during biogenesis, and potentially allow BamA to even tailor the folding of its diverse OMP substrates. Importantly, while the *E. coli* accessory proteins are not conserved across all Gram-negative bacteria, we hypothesize that other components in these bacteria would serve this role or that those BamA orthologs have evolved to self-regulate these conformational changes.

We also report the cryoEM structure of BAM/MBP-EspP^β9–12^, revealing a hybrid-barrel where the barrel of BamA opens to accommodate four strands of EspP. The first strand of the barrel of BamA pairs with the last strand of EspP, supporting the notion that the exposed edge of the first strand of BamA serves to initiate strand templating of the barrel of EspP. The C-terminal portion of the barrel of BamA appears to move mostly as a rigid body to accommodate the integration of the strands EspP. Interestingly, this region of the barrel contains the conserved loop 6 which interacts with the opposite inner wall of the barrel, thereby stabilizing this portion of the barrel and providing further explanation why disrupting this interaction has drastic effects on the function of BAM^18,61–63^. Further stabilization was observed by interactions between T6 and POTRA3, which may allow stabilized expansion of the barrel to accommodate integration of substrate OMPs during folding. In our low resolution structure, we also observe density trailing down from the four integrated EspP strands to the underside of BAM where additional density is observed, indicating that substrate OMPs are likely threaded up through the periplasmic side of BAM to the lateral seam of BamA during biogenesis. Importantly, given the limited space and constriction imposed by the periplasmic region of BAM, such a pathway would negate the notion that BAM mediates the insertion of preformed or partially folded substrate OMPs into the membrane. While it remains possible that BAM may use multiple mechanisms for the biogenesis of OMPs with differing sizes and properties, the BAM/MBP-EspP^β9–12^ structure confirms that one of those mechanisms is through the formation of a hybrid-barrel where strands are likely integrated sequentially, as opposed to being inserted in a concerted method or as pre-folded OMPs. We postulate that the BAM/MBP-EspP^β9–12^ structure represents an early intermediate of OMP biogenesis, while the recent structure of BAM in complex with a substrate BamA represents a late intermediate^42^. Taken together, these structures support the budding mechanism in which substrates are funneled from under BAM to the lateral seam where they are integrated into the barrel of BamA via strand templating and β-augmentation.

Our studies here, along with other recent reports^42–44^, allow us to propose a revised mechanistic model (Supplementary Fig. 17) where BAM is in a metastable state (outward-open) in the membrane initially and is pushed to an active state (inward-open) by initial interaction with the substrate and pairing of its β-signal with the first strand of BamA. This agrees with our BAM/MBP-EspP^β9–12^ structure where BAM is found in a state more closely matching inward-open. The substrate OMP barrel would then continue to fold and grow in this state until eventually budding out away from the BamA barrel, with the growing edge of the substrate OMP barrel being stabilized by the C-terminal region of the BamA barrel (along loop 6). Towards the later stages of biogenesis, BAM would then flip back to the outward-open state in order to accommodate the larger substate OMP barrel. This is supported by the recent structure of BAM in complex with a substrate BamA, which was observed in an outward-open state as a late-stage intermediate^42^. OMP biogenesis would then be completed once the first strand of the new OMP enters the lateral seam, leading to strand exchange from BamA, pairing of the first and last strands of the new OMP, and diffusion into the outer membrane.

## Methods

### Expression and purification of BAM

BAM was expressed and purified from *E. coli* as previously described^26,36^. Briefly, a single plasmid pJH114xB (pJH114^36^ containing an extra copy of BamB; Supplementary Table 2) containing all five Bam components was transformed into BL21(DE3) cells (NEB), plated onto LB-carbenicillin agar plate and incubated overnight at 37 °C. A single colony was used to prepare an overnight culture in 2xYT medium supplemented with 100 μg/ml ampicillin. The cells were centrifuged, resuspended, and used to inoculate larger cultures supplemented with 50 μg/ml ampicillin. At an OD_600_ between 0.8 and 1.0, the cells were induced with 0.2 mM IPTG and growth for an additional 3 h before harvesting. The cells were resuspended in 1x PBS containing 10 μg/ml DNaseI and 200 μM PMSF, and lysed by three passages through an Emulsiflex C-3 homogenizer (Avestin). The lysate was centrifuged at 6,000 × *g* for 20 min to remove cell debris, and the supernatant was centrifuged at 200,000 × *g* for 90 min at 4 °C to pellet the membranes. Membranes were resuspended in 1x TBS buffer containing 37 mM imidazole using a dounce homogenizer. Solubilized was performed by adding DDM to a final concentration of 0.5% with stirring at 4 °C for 4 h. Insoluble materials were removed by centrifugation at 200,000 × *g* for 40 min, and the supernatant collected.

BAM purification was performed using a HisTrap Nickel column (GE Healthcare) on a Pure L25 system (GE Healthcare) with an elution linear gradient of 37–500 mM imidazole with Buffer A (25 mM Tris-HCl, pH 7.5, 150 mM NaCl, 0.05% DDM, and 37 mM imidazole) and Buffer B (25 mM Tris-HCl, pH 7.5, 150 mM NaCl, 0.05% DDM, and 1 M imidazole). Fractions containing BAM were pooled, concentrated, and passed over a 16/60 Sephacryl S-300 HR column (GE Healthcare) at a flow rate of 1.0 ml/minute using 25 mM Tris-HCl, pH 7.5, 150 mM NaCl, 0.05% DDM. The peak fractions containing all five components of BAM were pooled and concentrated to ~100 μM.

### Reconstitution of the BAM complex into nanodiscs

Membrane scaffold proteins MSP1D1 (Addgene; gift from Stephen Sligar), MSP1E3D1 (Addgene; gift from Stephen Sligar), and MSP2N2 (kindly provided by J. Psonis) were expressed and purified as previously described^64^ (Supplementary Table 2), with the 6xHis tag being removed by TEV treatment. Nanodisc reconstitution was performed in a final volume of 300 μL by mixing the purified BAM complex, *E. coli* polar lipid (*E. coli* Polar Lipid Extract, Avanti Polar Lipids) and MSP protein in 1x TBS to a final concentration of 20 μM, 2 mM, and 100 μM, respectively. To initiate nanodisc reconstitution, washed Bio-beads SM2 resin (Biorad) was then added to the mixture to remove detergents and allowed to rock at 4 °C overnight. The supernatant containing the BAM nanodisc was then isolated using Ni-NTA resin (HisPur; ThermoFisher) and eluted with 25 mM Tris-HCl, pH 7.5, 150 mM NaCl and 400 mM imidazole. The elution containing the BAM nanodisc was loaded onto a Superdex 200 Increase 10/300 GL column (GE Healthcare) at a flow rate of 0.5 mL/minute with 25 mM Tris-HCl, pH 7.5 and 150 mM NaCl. The peak fractions for the BAM nanodisc were pooled.

### Preparation of BAM-inserted MSP nanodiscs from native membranes

A protocol to constitute a BAM in nanodiscs prepared from native membranes was developed to study the structure of BAM in a more native environment. The expression of BAM and lysis of cells were following the same procedures as above. After cell lysis, the lysate was centrifuged at 6000 × *g* for 20 min to remove cell debris and the supernatant collected. Triton X-100 was then added into the supernatant to a concentration of 2% to remove the inner membrane. The outer membrane containing the BAM complex was then pelleted by ultracentrifugation at 200,000 × *g* for 90 min at 4 °C. Using a dounce homogenizer, the membrane pellet was solubilized in 1x TBS solution containing MSP1E3D1 without His-tag and allowed to stir overnight. A BAM to MSP1E3D1 ratio of 1:4 was maintained in order for maximize the reconstitution efficiency of the BAM-inserted nanodiscs using native membranes. The next day, Ni-NTA resin (HisPur; ThermoFisher) was added to the solution and allowed to incubate for 8 h and the BAM nanodiscs were then eluted with 25 mM Tris-HCl, pH 7.5, 150 mM NaCl, and 400 mM imidazole. The elution containing the native BAM nanodisc was further purified using a Superdex 200 Increase 10/300 GL column at a flow rate of 0.5 mL/minute with 25 mM Tris-HCl, pH 7.5 and 150 mM NaCl. Peak fractions were pooled and concentrated for a second SEC to clean it up further. The peak fractions from the second SEC containing the BAM nanodiscs were pooled for cryoEM studies and lipidomics analysis.

### Lipidomics analysis

To confirm that preparations contained *E. coli* lipids, the BAM-inserted nanodiscs prepared using native membranes were analyzed using LC-MS/MS analysis (Purdue Proteomics Facility) and the results summarized in Supplementary Fig. 5. For complete coverage, both positive and negative ionization MS modes were used. For HPLC-MS analysis, separations were performed on an Agilent 1290 UPLC system (Palo Alto, CA). The lipids were assayed using a Waters BEH C18 column (1.7 µm, 2.1 × 100 mm), with a mobile phase flow rate of 0.40 mL/minute. Mobile phase A was ddH2O and mobile phase B was 50% isopropanol and 50% acetonitrile. Both mobile phases contained 0.1% formic acid and 10 mM ammonium acetate buffer. Initial conditions were 65:35 A:B, held for 0.5 min, followed by a linear gradient to 20:80 at 5 min, then 0:100 at 10 min, with a hold to 15 min. Column re-equilibration was performed by returning to 65:35 A:B at 17 min and holding until 21 min.

The mass analysis was obtained using an Agilent 6545 Q-TOF mass spectrometer with ESI capillary voltage 3.5 kV, nitrogen gas temperature 320 °C, drying gas flow rate 8.0 L/minute, nebulizer gas pressure 35 psig, fragmentor voltage 135 V, skimmer 465 V, and OCT RF 750 V. Sample was evaluated in both positive and negative ionization modes. MS data scans (from m/z 100–1200) were collected using Agilent MassHunter Acquisition software (v. B.06). Mass accuracy was improved by infusing Agilent Reference Mass Correction Solution (G1969-85001). MS/MS was performed in a data-dependent acquisition mode. Peak deconvolution was performed using Agilent Qualitative Analysis (v. B.06). Peak annotations were performed using the METLIN (metlin.scripps.edu) metabolite database, with a mass error of less than 10 ppm. Identifications were aided by MS/MS spectra comparisons.

### Cloning and expression MBP-EspP

MBP-EspP has been previously reported to be able to stall with BamA^41^. Taking advantage of this construct design, MBP-EspP (residues 748–1300 of EspP) with an N-terminal 6xHis tag was subcloned from pMAL-p5X (NEB) and pC6H1^32^ (kindly provided by Drs. Susan Buchanan and Travis Barnard) into the pHIS-Parallel2 vector (pHis2) (Supplementary Table 2). Expression of MBP- EspP was performed by transforming BL21(DE3) cells (NEB) with the pHis2/MBP-EspP vector. MBP-EspP was expressed as inclusion bodies and spun down at 5000 × *g* for 10 min after cell lysis. The inclusion bodies were then washed three times with 30 mL of 1x PBS containing 1% Elugent (Calbiochem) using a dounce homogenizer and then repelleted at 5000 × *g* for 10 min. The inclusion bodies were then washed three times with 30 mL of 1x PBS. The pellet was finally resuspended in 10 mL of 1x PBS, aliquoted and flash froze for future use.

### In vitro folding of MBP-EspP into the BAM nanodiscs

To reconstitute MBP-EspP into the BAM nanodisc, aliquot of MBP-EspP was first solubilized in 25 mM of Tris-HCl, pH 7.5 + 8 M Urea. In order to fold MBP-EspP into the BAM nanodisc, SurA was expressed and purified from *E. coli* as previously reported^59,65^. Briefly, the experiment was performed in a final volume of 500 μL by mixing the BAM nanodisc, SurA, MBP-EspP in 1x TBS containing 1 M urea to a final concentration of 1.8 μM, 25.2 μM, and 3.6 μM, respectively. The mixture was incubated overnight, gently spun and loaded directly onto a Superdex 200 Increase 10/300 GL column (GE Healthcare) at a flow rate of 0.5 mL/minute using 25 mM Tris-HCl, pH 7.5 and 150 mM NaCl. The peak fractions were then pooled and concentrated.

### Small-scale in vivo crosslinking

With the aim to improve the resolution for the structure of the BAM/EspP hybrid barrel, where we observed the stall of the last four strands from EspP with BAM, we performed in vivo crosslinking on a truncated EspP construct (Supplementary Table 2). A pRW1 vector was first generated by using the pCDF-1b vector (Novagen) as the backbone and swapping the T7 promotor region with araBAD promotor region from pBAD/HisA, to generate a vector that is streptomycin resistant with a pBAD promoter. MBP-EspP from the pHis2/MBP-EspP vector was first subcloned into the pRW1 vector to generate pRW1/MBP-EspP, the MBP tag was kept while the 6xHis tag was removed. Using pRW1/MBP-EspP as the template, the pRW1/MBP-EspP^β9–12^ containing the deletion of the first 8 strands from EspP was created via Golden Gate Assembly. Subsequently, the mutations including G1226C, G1228C, Q1230C, D1232C, and L1234C were introduced into pRW1/MBP-EspP^β9–12^, generating pRW1/MBP-EspP^G1226C_β9–12^, pRW1/MBP-EspP^G1228C_β9–12^, pRW1/MBP-EspP^Q1230C_β9–12^, pRW1/MBP-EspP^D1232C_β9–12^, and pRW1/MBP-EspP^L1232C_β9–12^, respectively. The plasmid pJH114 was also modified with C690S and C700S substitutions to create a cysteine-free version, which was further substituted with I806C or G807C to create pJH114^C690S/C700S^ with a single cysteine in β16 of BamA. BL21(DE3) cells containing the single cysteine mutant of pJH114 ^C690S/C700S^ and pRW1/MBP-EspP^β9–12^ mutants were grown at 37 °C, supplemented with 100 μg/mL of ampicillin and 50 μg/mL streptomycin until OD_600_ of ~0.6. Induction was first initiated with 0.2 mM IPTG for 30 min, and then 0.1 % (w/v) L-(+)-arabinose for an additional 4 h. The cells were then harvested and the crosslinked adducts were detected by western blot analysis as a higher molecular weight species.

### Large-scale expression, crosslinking, and purification of BAM/MBP−EspP^β9–12^ complex

Based on the small-scale results, we were able to purify the crosslinked adducts for BAM/MBP-EspP^β9–12^ using pJH114 ^C690S/C700S/G807C^ and pRW1/MBP-EspP^G1226C_β9–12^. The structure was solved, however, only the density for the last strand of EspP was resolved while the other strands were still mostly unresolved. We then reasoned that a single mutation at I806C on BamA could help stabilize the complex further, since I806C is pointing to the outside of the barrel while G807C is pointing into the lumen of the barrel structurally. Hence, the cysteine-free version of plasmid pJH114 with was further substituted with I806C to create pJH114^C690S/C700S/I806C^ for subsequent studies. BL21(DE3) cells containing pJH114 ^C690S/C700S/I806C^ and pRW1/MBP-EspP ^G1226C_β9–12^ (referred to EspP^β9–12^ within the text and figures) were grown overnight at 37 °C, supplemented with 100 μg/mL of ampicillin and 50 μg/mL streptomycin. The overnight culture was then inoculated into 4 × 500 mL of 2xYT medium containing 100 μg/mL of ampicillin and 50 μg/mL streptomycin until OD_600_ of ~0.6. Induction was first initiated with 0.2 mM IPTG for 30 min, and then 0.1 % (w/v) L-(+)-arabinose for an additional 4 h. Cells were then harvested and resuspended into 1x PBS and incubated with 0.3 mM dichloro(1,10-phenanthroline)copper(II) for 1 h to enhance the disulfide crosslinking. The cells were then pelleted again for subsequent purification.

The lysis and purification of BAM/MBP-EspP^β9–12^ complex was conducted as described previously here using a Ni-NTA column. Peak fractions after the initial stage of Ni-NTA purification were pooled for further purification using an MBPTrap column, eluted with 25 mM Tris-HCl, pH 7.5, 150 mM NaCl, 0.01% LMNG, and 10 mM maltose.

### EM data acquisition and analysis

For negative staining, 2.5 μL of purified BAM nanodisc (BAM_D1, BAM_E3, BAM_N2, BAM_E3HR, BAM_Native, and BAM_EspP) at a concentration of 0.06–0.08 mg/mL was applied to a glow-discharged grid with a thin layer of continuous carbon film (CF400-CU, electron microscopy sciences) and stained with uranyl formate (0.75%, w/v) as previously described^66^. The grids were then imaged using a Tecnai T20 TEM electron microscope (FEI) operated at 200 kV. Images were recorded using a CCD camera (Gatan US1000 2 k × 2k) with a defocus set to 1.5 μm, corresponding to a pixel size of 2.4 Å.

For cryoEM, 2.5 μL of purified complex-inserted nanodisc samples at a concentration of 0.6–0.8 mg/mL was applied to a glow-discharged Quantifoil grid (R3.5/1400 mesh), incubated for 30 s, and blotted using Vitrobot Mark IV (ThermoFisher) for 2 s with a blotting force of 2 at 4 °C and 100% humidity, which was then plunge froze in liquid ethane. For BAM_D1, BAM_E3, and BAM_N2, cryoEM data were collected on a Titan Krios microscope (ThermoFisher) operated at 300 kV with a nominal magnification of 81,000x using a K2 direct electron detector (Gatan) operated in super-resolution counting mode using Leginon^67^ for automated data collection. The images were recorded at a defocus range of 1.5 to 2.5 μm, with a calibrated physical pixel size of 0.69 Å/pixel. With 60 frames recorded, the total exposure time was 12 s, leading to a total dose rate of 43.85 e-/Å^2^.

CryoEM data on BAM_E3HR were collected on a Titan Krios microscope (ThermoFisher) operated at 300 kV with a nominal magnification of 81,000x using a K3 direct electron detector (Gatan) operated in super-resolution counting mode using Leginon^67^ for automated data collection. The images were recorded at a defocus range of 1.5 to 2.5 μm, with a calibrated physical pixel size of 0.525 Å/pixel. With 40 frames recorded, the total exposure time was 2.6 s, leading to a total dose rate of 44.9 e-/Å^2^. CryoEM sample preparation and data acquisition on BAM-inserted nanodiscs prepared from native membranes and BAM nanodisc with MBP − EspP followed the same procedure as above. For the high-resolution BAM/EspP^β9–12^ structure in LMNG, the samples were concentrated to ~2.0 mg/mL and blotted following the same protocol as above. Data collection parameters for the reported data in this study are summarized in table S1.

### Image processing

For analysis of the negative-stain images, the defocus of the EM images for BAM_D1 were determined using CTFFIND4^68^ followed by particle picking and image processing in RELION-2^69,70^. All the particles from a total of 15 images were picked, extracted, and processed for 2D classification. Projections used for projection matching were generated using CryoSPARC^71^.

For the cryoEM 3D reconstructions of BAM in different sized nanodiscs, a total of 961, 1115, and 1111 movies were collected for BAM_D1, BAM_E3, and BAM_N2, respectively. Motion correction on all the movie frames was conducted using MotionCor2^72^ with a binning factor of 2 implemented within RELION-2^69^. Contrast transfer function (CTF) on motion corrected sums were determined using CTFFIND4^68^. A total of 2000 particles were manually picked for each dataset to generate 2D class averages that had clearly defined and recognizable features for template-based particle picking. A total of 783,635 particles, 813,270 particles and 1,196,641 particles were autopicked and extracted from each dataset for BAM_D1, BAM_E3, and BAM_N2, respectively. These particles were subjected to further 2D classifications, and the remaining particles within the 2D class averages that had clearly defined and recognizable BAM features were combined for 3D classification. A remaining of 39,504 particles, 73,038 particles and 72,244 particles were selected and refined, resulting in reconstructions at 8.0, 6.9, and 7.5 Å resolution for BAM_D1, BAM_E3, and BAM_N2, respectively.

For the reconstruction of BAM in nanodiscs prepared from native membranes, a total of 2,247 movies were collected. Image processing was conducted using CryoSPARC^71^. Patch Motion Correction and Patch CTF Estimation were conducted followed by automated particle picking and 2D classification with a total of 54,989 particles. An ab initio model was generated and refined using Homogeneous Refinement to a resolution of 7.1 Å after FSC mask auto-tightening. Non-Uniform Refinement was used to produce a final map to 5.9 Å. For model building, we fit the BAM structure to the cryoEM map and performed real space refinement using PHENIX^73^.

For the high-resolution 3D reconstruction of BAM in E3 nanodiscs, motion correction on all the movie frames was conducted using MotionCor2^72^ with a binning factor of 2 implemented within RELION-3^69^. Contrast transfer function (CTF) on motion corrected sums were determined using GCTF^74^. For particle picking, reference free Laplacian-of-Gaussian auto-picking was conducted to generate initial 2D class averages. 2D class averages that had clearly defined and recognizable features were selected as the templates for template-based particle picking in RELION. A total of 8,053,211 particles were autopicked and extracted from 5317 dose-weighted micrographs. The dataset was split into eight parts for further 2D classification to select particles with good features, which was then combined for a second run of 2D classification. After this step, a remaining of 4,998,437 particles within the 2D class averages that had clearly defined and recognizable BAM features were combined for further processing. The dataset was further split into 10 parts for 3D classification, and each subset was classified into 8 classes. The particles from the good 3D classes were combined (1,121,059 particles) and refined in RELION with a soft mask, resulting in a 4.1 Å resolution reconstruction of BAM in nanodisc. 3D refinement after Bayesian polishing resulted in a map at 4.0 Å resolution.

A similar strategy was conducted to reconstruct the low resolution structure of BAM with MBP-EspP in nanodiscs using RELION-3. A total of 1,529,450 particles were picked and extracted from 1985 dose-weighted micrographs. A total of 263,098 particles were selected and classified in eight classes for 3D classification. The major class containing defined features of BAM with MBP-EspP (70,031 particles) was then refined using a soft mask in RELION to a resolution of 7.0 Å. For model building, the BAM structure from our 4.0 Å reconstruction was refined using MDFF^75^ and manually modified to incorporate four C-terminal strands of EspP. Both three and four strands were tested for EspP, however, four strands fit the strand constraints better than three. Further, with four strands, β9 was pointing directly into the periplasmic side of the barrel, exactly where additional density was observed trailing down into the large globular density on the periplasmic side of BAM. We then fit this extra density with a poly-alanine model, building just enough to enter the globular density sitting under BAM. While this globular density resembled a partial MBP molecule, we chose not to model it given the uncertainty. A similar pipeline was followed for the high resolution structure of BAM with MBP-EspP^β^9–12, where a total of 955,894 particles were picked and extracted from 4,405 dose-weighted micrographs. A total of 463,125 particles were selected and classified into 6 classes for 3D classification. One major class with defined features containing 149,420 particles were then selected, refined in CryoSPARC to a final resolution of 3.4 Å resolution.

All model building was performed using COOT^76^ and real space refinement performed for all structures using PHENIX^73^. Refinement parameters for the reported cryoEM structures in this study are summarized in table S1.

### Simulations of expected nanodisc density size

A ring with the user-specified diameter (11, 13, and 17 nm) was generated to simulate the outer boundary of a nanodisc. 5000 copies were made with random shifts in x and y directions and averaged into a single map. The maximum shift was defined by subtracting the radius of the nanodisc with the radius of the BAM core (estimated diameter of 40 Å) and a 10-angstrom mask. The script for these simulations can be accessed from the following URL: https://gist.github.com/wjiang/fef38d342ca51b7e89d282c653ddd1fa.

### Cloning and mutagenesis of BamA mutants

Mutations of *Ec*BamA were made following a standard site-directed mutagenesis protocol with slight modification using a pRSF1b vector containing wild-type BamA with a 10x HIS-tag^18,30,54^ as a template (primer sequences are available upon request). Disulfide mutants were made using a cysteine-less BamA pRSF1b construct (C2S: C690S,C700S) as a template. Notably, this cysteine-less BamA mutant behaves identical to wild-type^18,77^.

### Complementation assay of BamA mutants in JCM166 cells

Plasmids harboring BamA mutants under kanamycin selection were transformed into an *E.coli* BamA depletion strain JCM166 in which endogenous BamA is under control of an arabinose promoter^11^. Mutant effects were analyzed by comparison of cell growth on LB-agar plates. For disulfide crosslinking mutants, transformed cells were streaked on LB plates supplemented with 50 μg/mL kan in the presence or absence of 0.1% arabinose, and in the presence or absence of 15 mM TCEP. For the POTRA5-T4 disulfide mutant, the LB-agar plate was further supplemented with 100 μg/mL carbenicillin to mimic conditions for analyzing this mutant as previously reported^27^. Titered spot plate assays and susceptibility to antibiotics were used to further analyze growth defects of mutants that grow similar to wild type under normal conditions (LB + kan). Following transformation, cells were plated on LB agar in the absence of arabinose, 50 μg/mL kan, and grown overnight at 37 °C. Single colonies were used to inoculate 5 ml LB-kan cultures which were grown at 37 °C, shaking at 180 RPM, until OD_600_ reached ~1.5. Cells were then harvested by centrifugation, washed 3x with fresh LB, and concentrations of samples normalized to OD_600_ = 1.0. Cells were serial diluted as indicated and 4 μl of each dilution were spot plated onto LB-kan plates in the presence or absence of 0.1% arabinose. Where indicated, plates were further supplemented with vancomycin or rifampicin (75 μg/ml and 3 μg/ml, respectively). After plating cells, all plates were incubated at 37 °C for approximately 14 h followed by imaging. All assays were performed at least in triplicate with representative images shown in the figures.

### Western blot analysis

Western blots were performed using an IBlot2 transfer system (Life Technologies) and the following antibodies at indicated dilutions: αBamA (1:10,000), αMBP (1:5000; NEB), αHIS-HRP (1:7000; Millipore Sigma), αDegP-MBP(1:5000; kindly provided by J. Beckwith), αLamB (1:1000; kindly provided by R. Misra), αSurA (1:10,000; kindly provided by R. Misra), and polyclonal antibody that recognizes OmpA, OmpF, and OmpC (1:8000; kindly provided by R. Misra). With the exception of blots where only αHIS-HRP antibody was used, all other blots additionally used the secondary goat anti-rabbit or anti-mouse antibodies conjugated to HRP (1:15,000; Millipore Sigma). Blots were probed sequentially with different antibodies, although samples were also each probed individually to ensure non-reactivity between antibodies used. Immunoblots were visualized with the ECL Prime Kit (GE Healthcare) and imaged with an ImageQuant LAS 4000 Imaging System (GE Healthcare). All assays/blots were performed at least in triplicate with representative images shown in the figures.

### Analysis of protein levels in BamA mutant background

To assess how protein levels are affected by BamA mutants in JCM166 cells under endogenous BamA depleting conditions, cultures were grown in the absence of arabinose. For each mutant, a starter culture was grown in LB media, with 50 μg/mL kanamycin and in the absence of arabinose. The next day all culture densities were similar, and each was diluted to OD_600_ ~0.01 and then allowed to grow until OD_600_ ~1.5. Culture samples were then harvested by centrifugation and re-suspended in 1x PBS with concentrations normalized to OD_600_ ~5.0. Samples were mixed with SDS loading buffer, heated at 95 °C for 5 min and used for SDS-PAGE followed by immunoblotting. All assays/blots were performed at least in triplicate with representative images shown in the figures.

### Disulfide crosslink mutant analysis

To verify formation of disulfide crosslink mutants, JCM166 cells transformed with BamA disulfide mutants were grown in LB + 0.1% arabinose. After culture growth reached OD_600_ ~1.5, cells were pelleted, resuspended in 1 × PBS with concentrations normalized to OD_600_ ~5.0, and SDS-PAGE sample loading buffer added with and without 50 mM DTT. Samples were ran on a 5% SDS-PAGE gel, followed by immunoblotting and probing as described above with αHIS-HRP monoclonal antibody. All assays/blots were performed at least in triplicate with representative images shown in the figures.

### Proteinase K analysis

To evaluate cell surface exposure and conformational differences of *Ec*BamA disulfide mutants in vivo, proteinase K digestion assays were performed on whole cells as described previously^30,77^ with some modifications. For disulfide mutants, single colonies from LB + 0.1% arabinose plates were used to inoculate 5 ml LB-kan cultures supplemented with 0.01% arabinose. For other mutants, both plates and cultures were grown in the absence of arabinose. All cultures were grown at 37 °C shaking at 180 RPM for several hours until OD_600_ reached approximately 1.5. Samples were then harvested by centrifugation and re-suspended in cold 1x PBS with concentrations normalized to OD_600_ ~5.0. Proteinase K was then added to each sample (0.5 mg/ml) followed by incubation at 37 °C for 30 min. To stop the reaction, PMSF was added to a final concentration of 5 mM and incubated at room temperature for 5 min. Cells were centrifuged again, supernatant removed, and cells re-suspended in 1x PBS. SDS-PAGE loading dye containing DTT was added to each sample, followed by boiling and SDS-PAGE. Western blots were then performed as described above. All assays/blots were performed at least in triplicate with representative images shown in the figures.

### Molecular dynamics simulations

For simulations of the BAM complex and its derivatives, we initially constructed three systems using the tools in Visual Molecular Dynamics (VMD)^78^. First, starting from the BAM(in) structure in PDB 5D0O/pdb], we filled in missing pieces, including much of BamC, from other structures (PDBs 5D0Q, 5AYW, and 5EKQ). Critically, the terminal W810 residue and the disulfide bond between residues C690 and C700 in BamA were added. The BAM(out) and BAMΔΒ(out) structures were constructed in a similar manner starting from those in PDBs 5LJO and 5D0Q, respectively. Other systems were constructed starting from the BAM(in) model and deleting each accessory protein, leading to BAMΔΒ(in), BAMΔC(in), BAMΔD(in), and BAMΔE(in) systems.

Each BAM complex was placed in a model of the outer membrane (OM). The outer leaflet of the OM is made exclusively of 104 lipopolysaccharides, each composed of lipid A and ten sugars representing K12 *E. coli*^79^. For the inner leaflet, a simplified model was used, which contained 234 POPE lipids and 77 POPG lipids; we have previously shown that this simplified model behaves similarly to a more complex one with six different lipid types^80^. In all systems, lipoproteins BamB, BamC, BamD, and BamE were lipidated at their N-terminal cysteine, with the tri-acyl lipid anchor inserted into the inner leaflet of the OM. All systems were solvated with TIP3P water^81^. A mixture of Mg^2+^ and Ca^2+^ ions were added to neutralize the high negative charge on the LPS molecules. Additional K^+^ and Cl^-^ ions were added to the bulk water at a salt (KCl) concentration of 150 mM. All final system sizes are nearly 400,000 atoms.

After an initial equilibration using NAMD^82^, all systems were run using Amber16^83^ for 6 μs in triplicate. The CHARMM36 force field for proteins^84^ and lipids^85^ was used. A 12-Å cut-off for Lennard-Jones interactions was used along with a force-based switching function starting at 10 Å. Long-range electrostatics were calculated using the particle mesh Ewald method^86^. The temperature was maintained at 310 K and the pressure at 1 atm using Langevin dynamics and a Monte Carlo barostat, respectively. A uniform 4-fs time step was used by employing Hydrogen Mass Repartitioning^87,88^.

For rotation angle measurements, each angle was measured between two vectors in the plane of the membrane, each centered on the BamA β-barrel (carbonyl carbons of residues 425 to 810). The end-point of the first vector is the geometric center of all carbonyl carbons in BamB, C, D, and E in the BAM(in) system at the start of the simulation; this defines our 0° reference. For systems lacking one of the accessory proteins, the definition of the reference was also changed accordingly, still using the BAM(in) system but discounting the same missing accessory protein. The end-point of the second vector is the geometric center of the carbonyl carbons of the accessory proteins that are present in the simulation.

### Statistics and reproducibility

All SDS-PAGE gels, Western blots, and plate assays shown are representative of at least two repeats with similar results. Similarly, all negative-stain EM and cryo-EM micrographs show are representative of the filtered micrographs used for further analysis based on particle distribution, contrast, CTF-resolution fit, and ice thickness.

### Reporting summary

Further information on research design is available in the Nature Research Reporting Summary linked to this article.

## Supplementary information

Supplementary Information

Reporting Summary

## Data Availability

CryoEM maps and models have been deposited into the Electron Microscopy Data Bank (EMDB) and Protein Data Bank (PDB) with the following accession numbers: BAM in D1 nanodiscs (EMD-24476 and PDB ID 7RI7), BAM in N2 nanodiscs (EMD-24477 and PDB ID 7RI8), low-resolution BAM in E3 nanodiscs (EMD-24478 and PDB ID 7RI9), high-resolution BAM in E3 nanodiscs (EMD-24474 and PDB ID 7RI5), BAM in nanodiscs prepared from *E. coli* outer membranes (EMD-24475 and PDB ID 7RI6), low-resolution BAM/EspP (EMD- 24481 and PDB ID 7RJ5), and BAM/EspP^β9-12^ (EMD-24473 and PDB ID 7RI4). Plasmids and other non-commercially available reagents used in this study are available from N.N. under a material transfer agreement with Purdue University. Source data are provided with this paper and can be download from the journal website. Source data are provided with this paper.

## Source data

Source Data

## Code availability

The script for the simulations of expected nanodisc density size can be accessed from the following URL: https://gist.github.com/wjiang/fef38d342ca51b7e89d282c653ddd1fa.

## References

[1] Tommassen J (2010). Assembly of outer-membrane proteins in bacteria and mitochondria. Microbiology.

[2] Misra R (2012). Assembly of the beta-barrel outer membrane proteins in gram-negative bacteria, mitochondria, and chloroplasts. ISRN Mol. Biol..

[3] Rollauer, S. E., Sooreshjani, M. A., Noinaj, N. & Buchanan, S. K. Outer membrane protein biogenesis in Gram-negative bacteria. *Philos. Trans. R. Soc. Lond. B Biol. Sci.*10.1098/rstb.2015.0023 (2015). 10.1098/rstb.2015.0023 PMC4632599 26370935

[4] Schulz GE (2000). beta-Barrel membrane proteins. Curr. Opin. Struct. Biol..

[5] Schleiff E, Soll J (2005). Membrane protein insertion: mixing eukaryotic and prokaryotic concepts. EMBO Rep..

[6] Clantin B (2007). Structure of the membrane protein FhaC: a member of the Omp85-TpsB transporter superfamily. Science.

[7] Noinaj N (2012). Structural basis for iron piracy by pathogenic Neisseria. Nature.

[8] Ulrich T, Rapaport D (2015). Biogenesis of beta-barrel proteins in evolutionary context. Int. J. Med. Microbiol..

[9] Muhlenkamp M, Oberhettinger P, Leo JC, Linke D, Schutz MS (2015). Yersinia adhesin A (YadA)-beauty & beast. Int. J. Med. Microbiol..

[10] Maier T (2015). Conserved Omp85 lid-lock structure and substrate recognition in FhaC. Nat. Commun..

[11] Wu T (2005). Identification of a multicomponent complex required for outer membrane biogenesis in Escherichia coli. Cell.

[12] Wu R, Stephenson R, Gichaba A, Noinaj N (2020). The big BAM theory: An open and closed case? *Biochimica et biophysica acta*. Biomembranes.

[13] Ricci DP, Silhavy TJ (2012). The Bam machine: a molecular cooper. Biochim. Biophys. Acta.

[14] Hagan CL, Silhavy TJ, Kahne D (2011). beta-Barrel membrane protein assembly by the Bam complex. Annu. Rev. Biochem..

[15] Webb CT, Heinz E, Lithgow T (2012). Evolution of the beta-barrel assembly machinery. Trends Microbiol.

[16] Heinz E, Lithgow T (2014). A comprehensive analysis of the Omp85/TpsB protein superfamily structural diversity, taxonomic occurrence, and evolution. Front. Microbiol..

[17] Noinaj N, Gumbart JC, Buchanan SK (2017). The beta-barrel assembly machinery in motion. Nat. Rev. Microbiol..

[18] Noinaj, N. et al. Structural insight into the biogenesis of beta-barrel membrane proteins. *Nature* **501**, 385–390 (2013). 10.1038/nature12521 PMC3779476 23995689

[19] Tamm LK, Hong H, Liang B (2004). Folding and assembly of beta-barrel membrane proteins. Biochim. Biophys. Acta.

[20] Gentle IE, Burri L, Lithgow T (2005). Molecular architecture and function of the Omp85 family of proteins. Mol. Microbiol..

[21] Walther DM, Rapaport D, Tommassen J (2009). Biogenesis of beta-barrel membrane proteins in bacteria and eukaryotes: evolutionary conservation and divergence. Cell. Mol. Life Sci..

[22] Zeth K (2010). Structure and evolution of mitochondrial outer membrane proteins of beta-barrel topology. Biochim. Biophys. Acta.

[23] Jiang JH, Tong J, Tan KS, Gabriel K (2012). From evolution to pathogenesis: the link between beta-barrel assembly machineries in the outer membrane of mitochondria and gram-negative bacteria. Int. J. Mol. Sci..

[24] Hohr, A. I. C. et al. Membrane protein insertion through a mitochondrial beta-barrel gate. *Science*10.1126/science.aah6834 (2018). 10.1126/science.aah6834 PMC5959003 29348211

[25] Diederichs KA (2020). Structural insight into mitochondrial beta-barrel outer membrane protein biogenesis. Nat. Commun..

[26] Bakelar J, Buchanan SK, Noinaj N (2016). The structure of the beta-barrel assembly machinery complex. Science.

[27] Gu Y (2016). Structural basis of outer membrane protein insertion by the BAM complex. Nature.

[28] Iadanza MG (2016). Lateral opening in the intact beta-barrel assembly machinery captured by cryo-EM. Nat. Commun..

[29] Han L (2016). Structure of the BAM complex and its implications for biogenesis of outer-membrane proteins. Nat. Struct. Mol. Biol..

[30] Noinaj N (2014). Lateral opening and exit pore formation are required for BamA function. Structure.

[31] Kim KH, Aulakh S, Paetzel M (2012). The bacterial outer membrane beta-barrel assembly machinery. Protein Sci..

[32] Barnard TJ, Dautin N, Lukacik P, Bernstein HD, Buchanan SK (2007). Autotransporter structure reveals intra-barrel cleavage followed by conformational changes. Nat. Struct. Mol. Biol..

[33] Ruiz-Perez F (2009). Roles of periplasmic chaperone proteins in the biogenesis of serine protease autotransporters of Enterobacteriaceae. J. Bacteriol..

[34] Ieva R, Tian P, Peterson JH, Bernstein HD (2011). Sequential and spatially restricted interactions of assembly factors with an autotransporter beta domain. Proc. Natl Acad. Sci. USA.

[35] Pavlova O, Peterson JH, Ieva R, Bernstein HD (2013). Mechanistic link between beta barrel assembly and the initiation of autotransporter secretion. Proc. Natl Acad. Sci. USA.

[36] Roman-Hernandez G, Peterson JH, Bernstein HD (2014). Reconstitution of bacterial autotransporter assembly using purified components. Elife.

[37] Barnard TJ (2012). Molecular basis for the activation of a catalytic asparagine residue in a self-cleaving bacterial autotransporter. J. Mol. Biol..

[38] Bernstein, H. D. Type V secretion in gram-negative bacteria. *EcoSal Plus*10.1128/ecosalplus.ESP-0031-2018 (2019). 10.1128/ecosalplus.esp-0031-2018 PMC6404772 30838971

[39] Leo JC, Grin I, Linke D (2012). Type V secretion: mechanism(s) of autotransport through the bacterial outer membrane. Philos. Trans. R. Soc. Lond. B Biol. Sci..

[40] Meuskens I, Saragliadis A, Leo JC, Linke D (2019). Type V secretion systems: an overview of passenger domain functions. Front. Microbiol..

[41] Doyle MT, Bernstein HD (2019). Bacterial outer membrane proteins assemble via asymmetric interactions with the BamA beta-barrel. Nat. Commun..

[42] Tomasek D (2020). Structure of a nascent membrane protein as it folds on the BAM complex. Nature.

[43] Iadanza MG (2020). Distortion of the bilayer and dynamics of the BAM complex in lipid nanodiscs. Commun. Biol..

[44] Xiao L (2021). Structures of the beta-barrel assembly machine recognizing outer membrane protein substrates. FASEB J..

[45] Malinverni JC (2006). YfiO stabilizes the YaeT complex and is essential for outer membrane protein assembly in Escherichia coli. Mol. Microbiol..

[46] Lee J (2018). Substrate binding to BamD triggers a conformational change in BamA to control membrane insertion. Proc. Natl Acad. Sci. USA.

[47] Robert V (2006). Assembly factor Omp85 recognizes its outer membrane protein substrates by a species-specific C-terminal motif. PLoS Biol..

[48] Imai Y (2019). A new antibiotic selectively kills Gram-negative pathogens. Nature.

[49] Luther A (2019). Chimeric peptidomimetic antibiotics against Gram-negative bacteria. Nature.

[50] Hart EM (2019). A small-molecule inhibitor of BamA impervious to efflux and the outer membrane permeability barrier. Proc. Natl Acad. Sci. USA.

[51] Sinnige T (2015). Conformational plasticity of the POTRA 5 domain in the outer membrane protein assembly factor BamA. Structure.

[52] Sinnige T (2015). Insight into the conformational stability of membrane-embedded BamA using a combined solution and solid-state NMR approach. J. Biomol. NMR.

[53] Liu J, Gumbart JC (2020). Membrane thinning and lateral gating are consistent features of BamA across multiple species. PLoS Comput. Biol..

[54] Lundquist K, Bakelar J, Noinaj N, Gumbart JC (2018). C-terminal kink formation is required for lateral gating in BamA. Proc. Natl Acad. Sci. USA.

[55] Hartmann JB, Zahn M, Burmann IM, Bibow S, Hiller S (2018). Sequence-specific solution NMR assignments of the beta-barrel insertase BamA to monitor its conformational ensemble at the atomic level. J. Am. Chem. Soc..

[56] Fleming PJ (2016). BamA POTRA domain interacts with a native lipid membrane surface. Biophys. J..

[57] Sinnige, T. et al. Solid-state NMR studies of full-length BamA in lipid bilayers suggest limited overall POTRA mobility. *J. Mol. Biol*. 10.1016/j.jmb.2014.02.007 (2014). 10.1016/j.jmb.2014.02.007 24530687

[58] Vuong P, Bennion D, Mantei J, Frost D, Misra R (2008). Analysis of YfgL and YaeT interactions through bioinformatics, mutagenesis, and biochemistry. J. Bacteriol..

[59] Hagan CL, Kim S, Kahne D (2010). Reconstitution of outer membrane protein assembly from purified components. Science.

[60] Gunasinghe SD (2018). The WD40 protein BamB mediates coupling of BAM complexes into assembly precincts in the bacterial outer membrane. Cell Rep..

[61] Gentle I, Gabriel K, Beech P, Waller R, Lithgow T (2004). The Omp85 family of proteins is essential for outer membrane biogenesis in mitochondria and bacteria. J. Cell Biol..

[62] Tellez R, Misra R (2012). Substitutions in the BamA beta-barrel domain overcome the conditional lethal phenotype of a DeltabamB DeltabamE strain of Escherichia coli. J. Bacteriol..

[63] Leonard-Rivera M, Misra R (2012). Conserved residues of the putative L6 loop of Escherichia coli BamA play a critical role in the assembly of beta-barrel outer membrane proteins, including that of BamA itself. J. Bacteriol..

[64] Denisov IG, Grinkova YV, Lazarides AA, Sligar SG (2004). Directed self-assembly of monodisperse phospholipid bilayer Nanodiscs with controlled size. J. Am. Chem. Soc..

[65] Bitto E, McKay DB (2002). Crystallographic structure of SurA, a molecular chaperone that facilitates folding of outer membrane porins. Structure.

[66] Booth, D. S., Avila-Sakar, A. & Cheng, Y. Visualizing proteins and macromolecular complexes by negative stain EM: from grid preparation to image acquisition. *J. Vis. Exp*. 10.3791/3227 (2011). 10.3791/3227 PMC3369646 22215030

[67] Suloway C (2005). Automated molecular microscopy: the new Leginon system. J. Struct. Biol..

[68] Mindell JA, Grigorieff N (2003). Accurate determination of local defocus and specimen tilt in electron microscopy. J. Struct. Biol..

[69] Scheres SH (2012). RELION: implementation of a Bayesian approach to cryo-EM structure determination. J. Struct. Biol..

[70] Scheres SH (2012). A Bayesian view on cryo-EM structure determination. J. Mol. Biol..

[71] Punjani A, Rubinstein JL, Fleet DJ, Brubaker MA (2017). cryoSPARC: algorithms for rapid unsupervised cryo-EM structure determination. Nat. Methods.

[72] Zheng SQ (2017). MotionCor2: anisotropic correction of beam-induced motion for improved cryo-electron microscopy. Nat. Methods.

[73] Adams PD (2010). PHENIX: a comprehensive Python-based system for macromolecular structure solution. Acta Crystallogr. Sect. D Biol. Crystallogr..

[74] Zhang K (2016). Gctf: real-time CTF determination and correction. J. Struct. Biol..

[75] Trabuco LG, Villa E, Mitra K, Frank J, Schulten K (2008). Flexible fitting of atomic structures into electron microscopy maps using molecular dynamics. Structure.

[76] Emsley P, Lohkamp B, Scott WG, Cowtan K (2010). Features and development of Coot. Acta Crystallogr. Sect. D Biol. Crystallogr..

[77] Rigel NW, Ricci DP, Silhavy TJ (2013). Conformation-specific labeling of BamA and suppressor analysis suggest a cyclic mechanism for beta-barrel assembly in Escherichia coli. Proc. Natl Acad. Sci. USA.

[78] Humphrey, W., Dalke, A. & Schulten, K. VMD: visual molecular dynamics. *J. Mol. Graph* **14**, 33–38, 27–38 (1996). 10.1016/0263-7855(96)00018-5 8744570

[79] Balusek C, Gumbart JC (2016). Role of the native outer-membrane environment on the transporter BtuB. Biophys. J..

[80] Hwang H, Paracini N, Parks JM, Lakey JH, Gumbart JC (2018). Distribution of mechanical stress in the Escherichia coli cell envelope. Biochim. Biophys. Acta Biomembr..

[81] Jorgensen WL, Chandrasekhar J, Madura JD, Impey RW, Klein ML (1983). Comparison of simple potential functions for simulating liquid water. J. Chem. Phys..

[82] Phillips JC (2005). Scalable molecular dynamics with NAMD. J. Comput. Chem..

[83] Case DA (2005). The Amber biomolecular simulation programs. J. Comput. Chem..

[84] Best RB (2012). Optimization of the additive CHARMM all-atom protein force field targeting improved sampling of the backbone phi, psi and side-chain chi(1) and chi(2) dihedral angles. J. Chem. Theory Comput..

[85] Klauda JB (2010). Update of the CHARMM all-atom additive force field for lipids: validation on six lipid types. J. Phys. Chem. B.

[86] Darden T, York D, Pedersen L (1993). Particle mesh ewald - an N.Log(N) method for ewald sums in large systems. J. Chem. Phys..

[87] Hopkins CW, Le Grand S, Walker RC, Roitberg AE (2015). Long-time-step molecular dynamics through hydrogen mass repartitioning. J. Chem. Theory Comput..

[88] Balusek C (2019). Accel erating membrane simulations with hydrogen mass repartitioning. J. Chem. Theory Comput..

