# Supplementary material for: Plasticity within the barrel domain of BamA mediates a hybrid-barrel mechanism by BAM
Source: Nat Commun. 2021 Dec 8;12:7131. doi: 10.1038/s41467-021-27449-4 (PMC8655018; doi:10.1038/s41467-021-27449-4)
Supplement: Supplementary file 1 — Supplementary Information [file 41467_2021_27449_MOESM1_ESM.pdf]

Supplementary Information for the following manuscript:

Plasticity within the barrel domain of BamA mediates a hybrid-barrel mechanism by BAM

Runrun Wu[1,*], Jeremy W. Bakelar[2,*,†], Karl Lundquist[3,*,¥], Zijian Zhang[3], Katie M. Kuo[4], David Ryoo[5], Yui Tik Pang[3], Chen Sun[2], Tommi White[6], Thomas Klose[2,7], Wen Jiang[1,2,7,8], James C. Gumbart[3,4,±], and Nicholas Noinaj[1,2,8,±]

[1] Interdisciplinary Life Science - PULSe, Purdue University, West Lafayette, Indiana, 47907

[2]Markey Center for Structural Biology, Department of Biological Sciences, Purdue University, West Lafayette, Indiana, 47907

[3]School of Physics, Georgia Institute of Technology, Atlanta, Georgia, 30332

[4]School of Chemistry and Biochemistry, Georgia Institute of Technology, Atlanta GA 30332

[5]Interdisciplinary Bioengineering Graduate Program, Georgia Institute of Technology, Atlanta, GA 30332

[6]Electron Microscopy Core Research Facility, W125 Vet Med Building, University of Missouri, Columbia, Missouri, 65211

[7]Purdue CryoEM Facility, Suite 171, Hockmeyer Hall for Structural Biology, Purdue University, West Lafayette, Indiana, 47907

[8]Purdue Institute of Inflammation, Immunology and Infectious Disease, Purdue University, West Lafayette, Indiana, 47907

Supplementary Figure 1. **Negative-stain EM of BAM-inserted nanodiscs. a**. BAM inserted into MSP1D1 (D1). **b**. BAM inserted into MSP1E3D1 (E3). **c**. BAM inserted into MSP2N2 (N2). For panels A-C, top row shows SEC traces along with an SDS-PAGE gel of the peak fraction, while the bottom row is a negative-stain EM micrograph using 0.75% uranyl formate. The scale bars represent 50 nm. Source data are provided as a Source Data file (same as in the main text Figure 1, panels c). **d**. Shown are 2D classes from 2D classification of BAM-inserted D1 nanodiscs, with projection matching (20 Å lowpass filtered), and model orientation matching based on the BAM structure in the outward-open state.

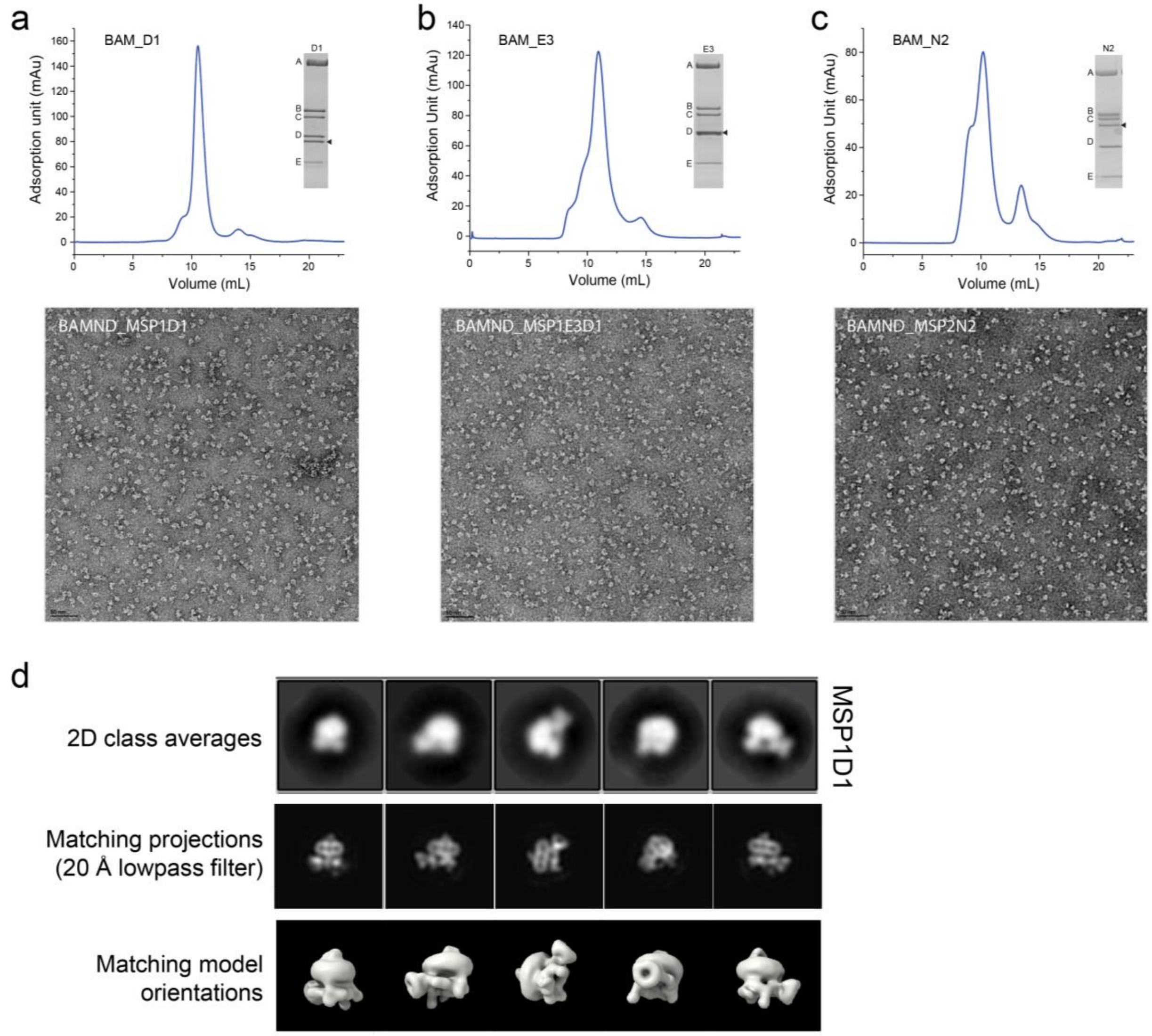

Supplementary Figure 2. CryoEM reconstructions of BAM in different sized nanodiscs. **a**. Shown are 2D class averages from 2D classification of BAM in MSP1D1, MSP1E3D1, and MSP2N2 nanodiscs. **b**. 3D reconstructions of BAM in each of the different sized nanodiscs, showing side, top, and bottom views. **c**. A superposition of the three reconstructions revealing nearly identical reconstructions, with the bottom panel showing minimal differences in the apparent sizes of the nanodisc densities. **d**. The BAM/MSP1D1 cryoEM map (transparent gray isosurface) is shown aligned to the outward-open (green) and inward-open (magenta) states of BAM. Overall fit to the map is shown on the left with a zoomed view of the lateral seam shown on the right. **e**. Orthogonal views of the superposition of the refined D1, E3, N2, and native membrane nanodisc structures; calculated RMSD values ranged from 1.5 – 2.2, indicating minimal conformational changes.

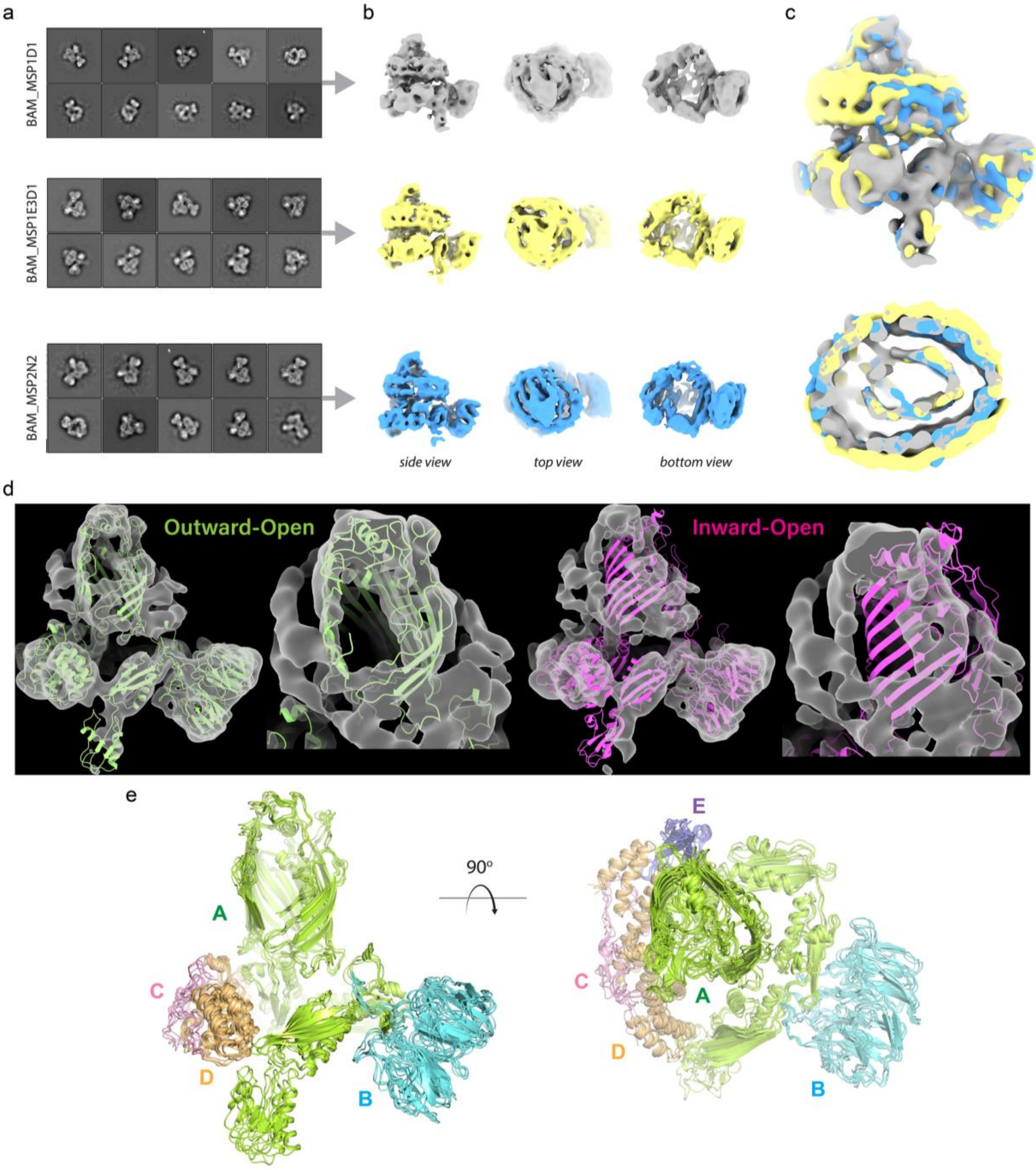

Supplementary Figure 3. High-resolution cryoEM reconstruction of BAM-inserted E3 nanodiscs. **a**. A representative motion corrected micrograph. The scale bar represents 100 nm. **b**. Selected 2D classes from 2D classification. **c**. Eight 3D classes from 3D classification. **d**. Refined 3D map to 4 Å resolution showing plots for angular distribution for all particles (**e**), FSC resolution estimation (**f**), and local resolution (**g**) of the final reconstruction.

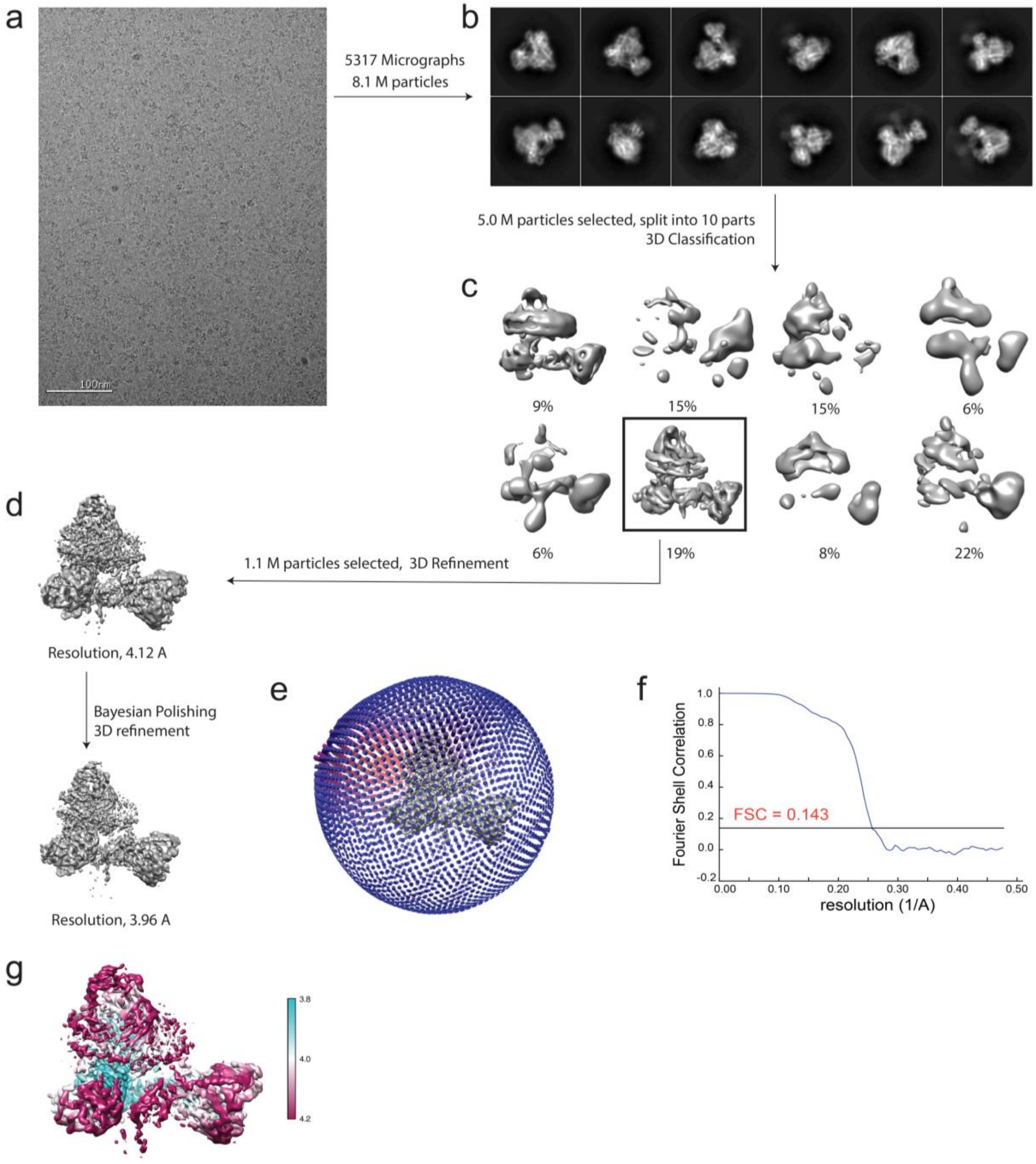

Supplementary Figure 4. **Simulations of BAM in different sized nanodiscs**. **a**. Despite using different sized nanodiscs, we observed nearly identical sized nanodisc densities for all of our reconstructions. To explain this, we rationalized that aligning and averaging a circular shaped object (representing BAM) randomly positioned within a ring (representing the nanodisc) would lead to averaging out of density for most of the volume except for the region along the outer perimeter of the object within the ring. **b**. To further verify our rationalization, we performed computational simulations of the three sizes of nanodiscs (11, 13, and 17 nm) with a randomly oriented 4 nm circular object (BAM). Upon averaging, a concentration of density with the highest intensity was indeed observed along the outer perimeter of the object. **c**. Measurements of the resulting densities for the different sized nanodiscs resulted in an average diameter of 8.5 nm with a range from 8.2 to 8.9 nm. These studies demonstrate that if objects within nanodiscs are randomly positioned, the observed density for the nanodisc in the 3D reconstruction will be dependent on the size of the object itself, assuming it is sufficiently large enough to dictate the 2D/3D alignments.

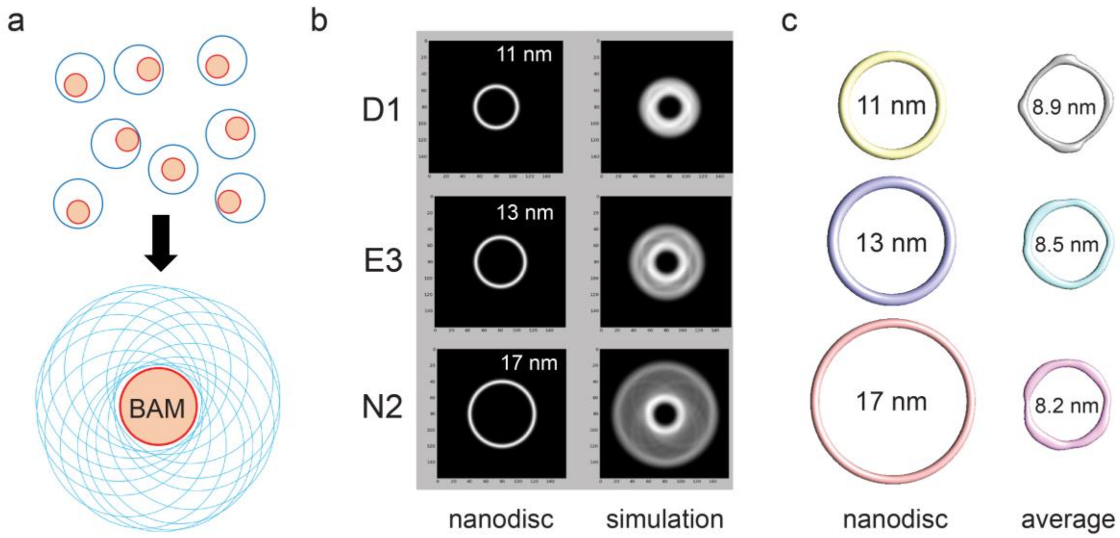

Supplementary Figure 5. **CryoEM structure of BAM-MSP nanodiscs prepared from native membranes**. **a**. SEC trace of BAM-inserted nanodiscs prepared from the outer membranes of BL21(DE3) cells. Source data are provided as a Source Data file (same as Figure 1, panel c). **b**. CryoEM micrograph and 2D class averages. **c**. The cryoEM reconstruction of BAM-MSP nanodiscs prepared from native membranes. **d**. Orthogonal views of the refined BAM structure fit into the cryoEM density map showing an outward-open conformation. **e**. The cryoEM map (transparent gray isosurface) is shown aligned to the outward-open (green) and inward-open (magenta) states of BAM. Overall fit to the map is shown on the left with a zoomed view of the lateral seam shown on the right. **f**. HPLC trace of the Blank (black) and BAM MSP nanodiscs from native membranes (red). **g**. Summary of selected hits (Lipid-A, PE, PG, and CL molecules) from the mass spec analysis with Scores ~80% or higher.

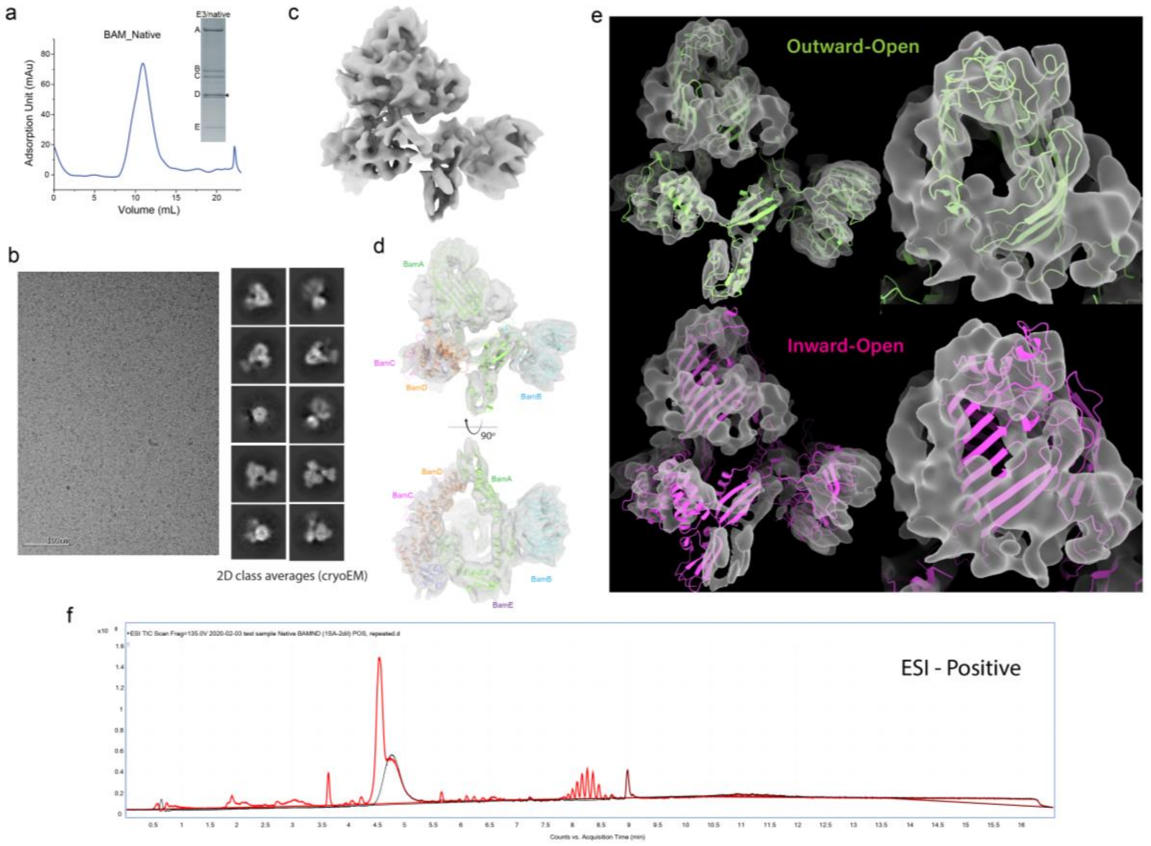

g

Name	Formula	Score (%)	Mass	Mass (DB)	LC Retention Time	METLIN ID#
Lipid A -disaccharide-1-P	C68 H129 N2 O20 P	94.19	1324.8931	1324.8876	5.689	41554
PE(10:0/13:0)[U]	C28 H56 N O8 P	77.64	565.3756	565.3744	4.593	40663
PE(12:0/13:0)[U]	C30 H60 N O8 P	97.87	593.4039	593.4057	4.609	40456
PE(16:0/P-16:0)	C37 H74 N O7 P	89.13	675.5206	675.5203	7.818	60379
PE(19:0/20:2(11Z,14Z))	C44 H84 N O8 P	88.79	785.6005	785.5935	7.874	77018
PE(19:0/20:5(5Z,8Z,11Z,14Z,17Z))	C44 H78 N O8 P	89.74	779.5468	779.5465	8.988	77021
PE(20:0/15:1(9Z))	C40 H78 N O8 P	89.36	731.5465	731.5465	8.989	77061
PE(20:0/P-18:1(11Z))	C43 H84 N O7 P	80.53	757.5927	757.5985	6.822	60644
PE(20:3(8Z,11Z,14Z)/17:2(9Z,12Z))	C42 H74 N O8 P	85.8	751.5214	751.5152	6.899	77147
PE(24:0/18:1(11Z))	C47 H92 N O8 P	82.71	829.6506	829.6561	7.384	61106
PE(24:1(15Z)/24:1(15Z))	C53 H102 N O8 P	88.89	911.7302	911.7343	8.354	61158
PE(O-16:0/21:0)	C42 H86 N O7 P	84.79	747.6083	747.6142	7.41	77513
PE(O-20:0/19:1(9Z))	C44 H88 N O7 P	85.23	773.6248	773.6298	7.321	77557
PE-Cer(d15:1(4E)/20:0(2OH))	C37 H75 N2 O7 P	90.08	690.5288	690.5312	7.506	103113
PE-NMe(20:0/20:0)	C46 H92 N O8 P	83.29	817.6498	817.6561	7.239	40741
PG(14:0/13:0)	C33 H65 O10 P	79.04	652.4323	652.4315	7.604	78918
PG(14:0/20:1(11Z))	C40 H77 O10 P	93.91	748.5238	748.5254	8.873	78927
PG(14:1(9Z)/18:0)	C38 H73 O10 P	94.49	720.4933	720.4941	8.403	78944
PG(15:0/14:0)	C35 H69 O10 P	88.17	680.4576	680.4628	8.988	78965
PG(16:0/13:0)	C35 H69 O10 P	79.03	680.4637	680.4628	8.154	79017
PG(16:1(9Z)/12:0)	C34 H65 O10 P	87.9	664.4377	664.4315	4.458	79026
PG(18:0/14:1(9Z))	C38 H73 O10 P	81.66	720.4924	720.4941	8.403	79134
PG(19:1(9Z)/13:0)	C38 H73 O10 P	88.19	720.4997	720.4941	8.989	79306
PG(O-16:0/12:0)	C34 H69 O9 P	78.59	652.4744	652.4679	6.077	79812
PG(O-20:0/20:3(8Z,11Z,14Z))	C46 H87 O9 P	82.13	814.6175	814.6088	8.271	79873
CL(20:3(5Z,8Z,11Z)/18:2(9Z,12Z)/18:2(9Z,12Z)/18:2(9Z,12Z))	C83 H144 O17 P2	88.87	1474.9835	1474.9879	5.608	61589
CL(20:4(5Z,8Z,11Z,14Z)/18:2(9Z,12Z)/18:2(9Z,12Z)/18:2(9Z,12Z))	C83 H142 O17 P2	93.4	1472.9719	1472.9722	6.102	61595
CL(20:4(8Z,11Z,14Z,17Z)/20:3(5Z,8Z,11Z)/18:2(9Z,12Z)/18:2(9Z,12Z))	C85 H144 O17 P2	79.73	1498.9847	1498.9879	6.074	61607

Supplementary Figure 6. Dynamics of BAM from all MD simulations. Dynamics of accessory proteins viewed from the extracellular side in the x-y (membrane) plane are shown in different colors with confidence ellipses (3σ). All data was aligned with the center of the BamA's β-barrel which is at (0, 0). Replica 1 (solid line), replica 2 (break line) and replica 3 (dotted line) are independent 6-μs simulations. The positions of the lipoproteins taken from refined experimentally determined structures of the inward-open (PDB 5D0O) and outward-open (PDB 5LJO) states are shown as squares and diamonds, respectively. Each panel shows the dynamics from a different initial state, BAM(in), BAMΔB(in), BAMΔC(in), BAMΔD(in), BAMΔE(in), BAM(out) and BAMΔB(out), separately.

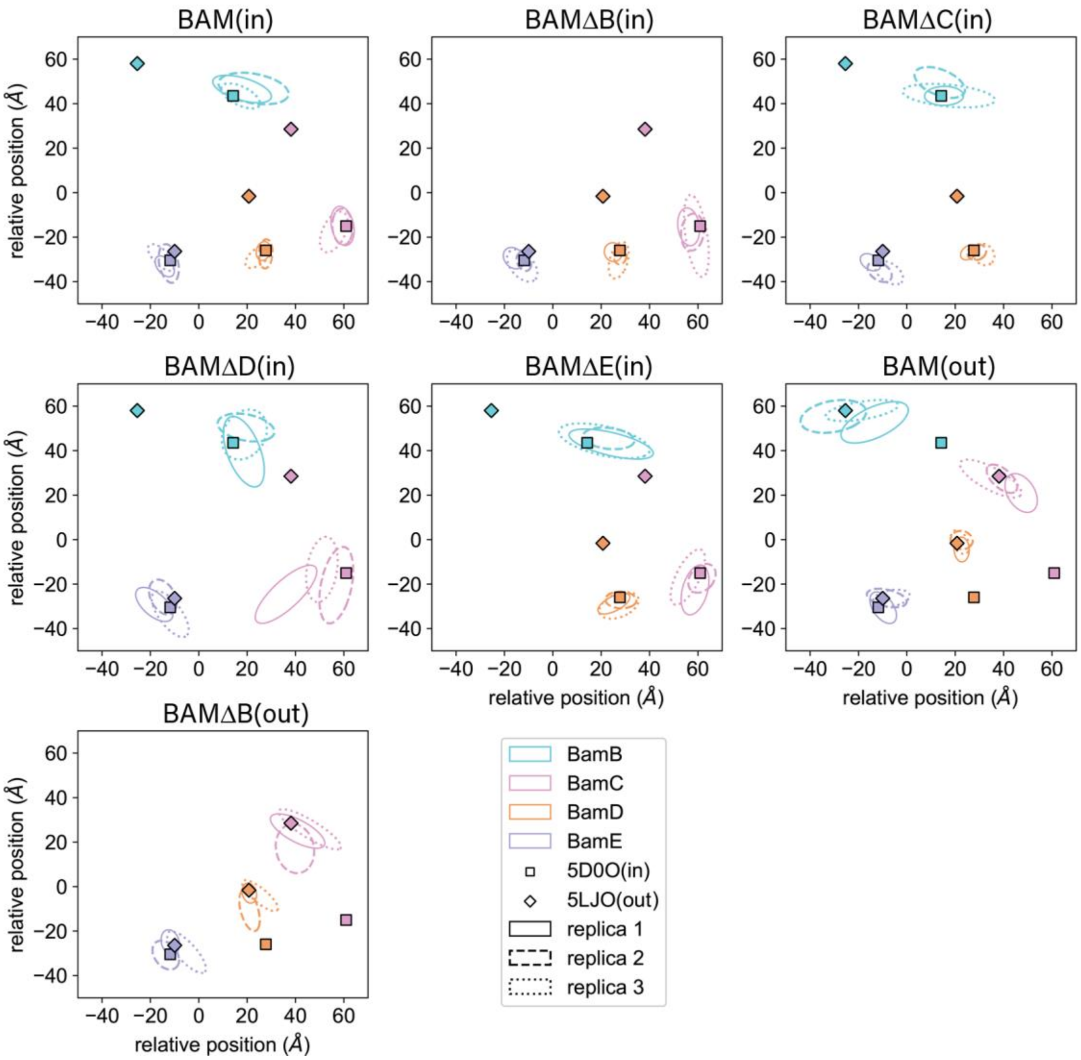

Supplementary Figure 7. **Rotation angle of the periplasmic domain ring in the membrane plane.** Rotation angles of the periplasmic domain ring in the membrane plane were calculated using the average angle made by BamB (when present), BamC and BamD with respect to BamA. Angles for systems BAMΔC(in) (**a**), BAMΔD(in) (**b**), and BAMΔE(in) (**c**) are shown. The angle of these lipoproteins in the structure 5D0O is used as a reference and is measured counterclockwise as viewed from the extracellular space. Plots are from three independent 6-μs simulations demonstrating a range of possible dynamics on this time scale. The dotted lines are the rotation angles in the structures used to initialize the inward-open state (0°; PDB 5D0O) and the outward-open state (63°; PDB 5LJO).

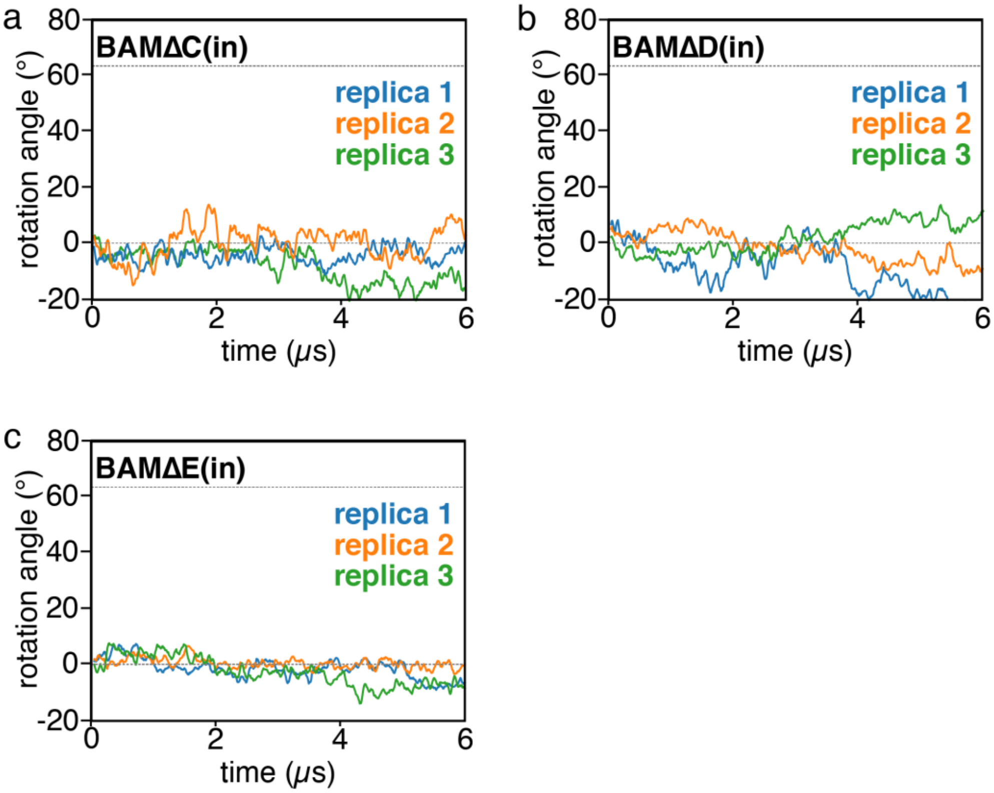

Supplementary Figure 8. Snapshots from the beginning (0 μs) and end (6 μs) of trajectories for the BAMΔB. a. BAMΔB(in), replica 3. **b**. BAMΔB(out), replica 3. **c**. BAMΔB(out), replica 2.

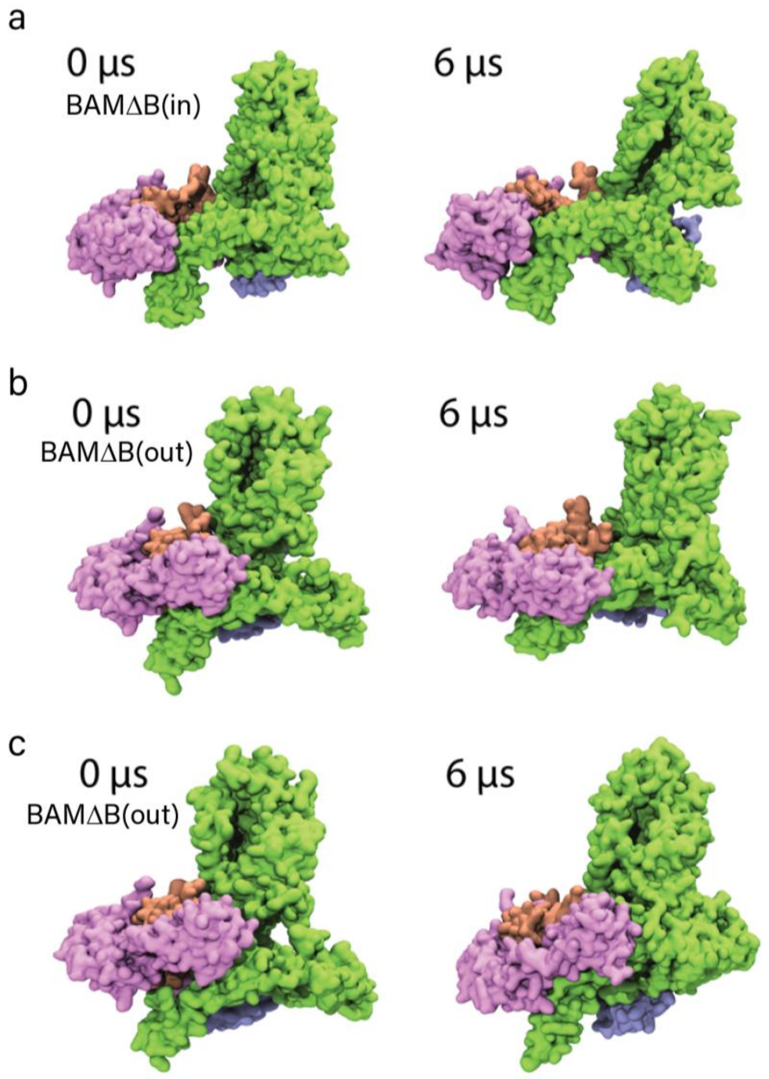

Supplementary Figure 9. Moving average of the hydrogen bonds between the backbones of BamA lateral seam strands for BAMΔC, BAMΔD, and BAMΔE. Each panel shows the result of each system as titled, with three lines representing the result of each individual simulation.

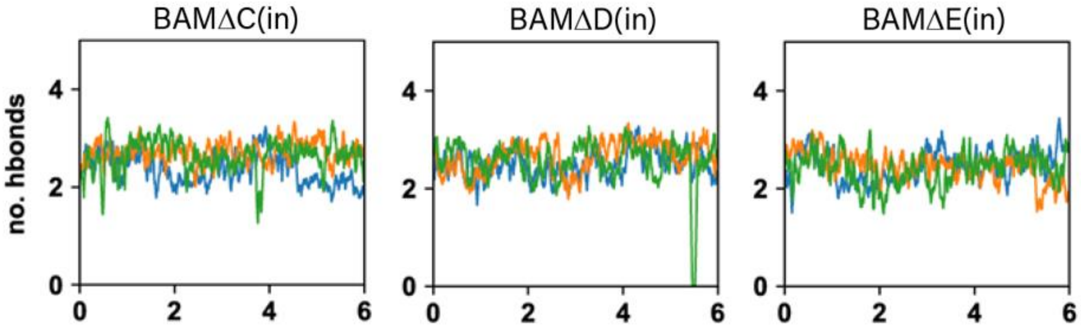

Supplementary Figure 10. Confidence ellipses based on x-y position of BamA's POTRA5 domain viewed from the extracellular side. All data was aligned with the center of the BamA's β-barrel which is at (0, 0). **a**. Ellipses for the combinations of all three 6-μs simulations are shown for the four indicated systems. **b**. Replica 1 (solid line), replica 2 (broken line) and replica 3 (dotted line) are independent 6-μs simulations. Different colors represent confidence ellipses based on different initial states. The positions of the POTRA5 taken from experimentally determined structures of the inward-open (PDB 5D0O) and outward-open (PDB 5LJO) states are shown as squares and diamonds, respectively.

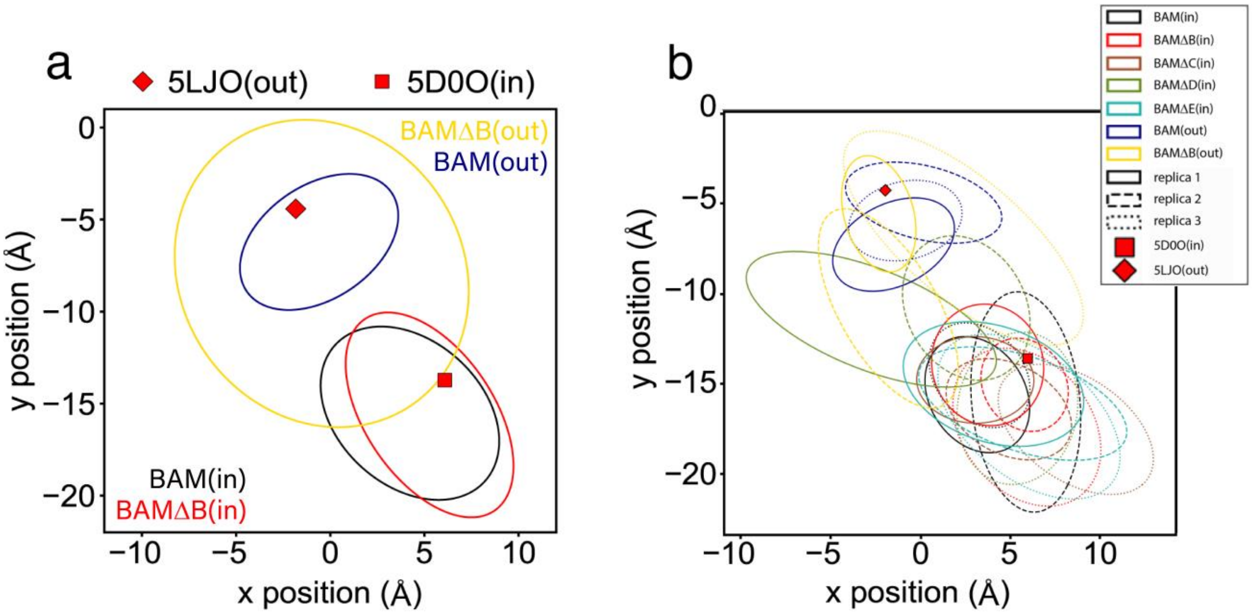

Supplementary Figure 11. **BamA loop 3 crosslink mutant screening**. A crosslink mutant, designed to lock loop 3 to loop 6 in an outward-open state, was further assayed using colony titer assays and challenged with vancomycin (vanco) and rifampicin (rif). No effect was observed in the absence of arabinose, with only minimal effects even in the presence of both vancomycin and rifampicin. Assays were performed at least in triplicate with a representative image shown.

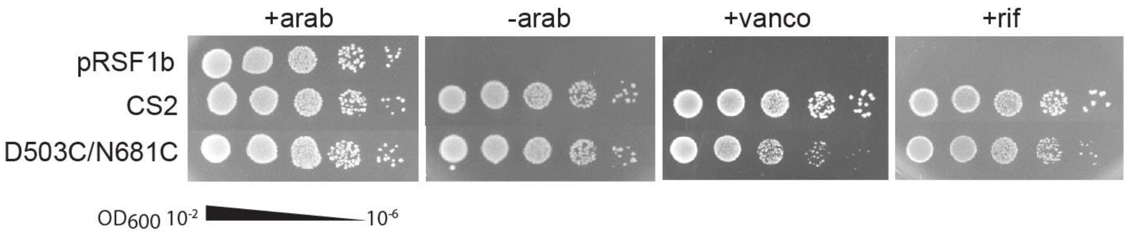

Supplementary Figure 12. **Targeted molecular dynamics (TMD) simulations of disulfide crosslink mutants**. Disulfide crosslink mutants include (**a**) 502C/706C, (**b**) 503C/681C, (**c**) 393C/584C, (**d**) 425C/808C and (**e**) 431C/807C. The left panel shows the target structure (in white) and the structure at the end of the 100-ns simulation. Green indicates that the mutation was non-lethal (b), while tan indicates a growth-defect only when challenged with antibiotics (c), and red lethal (a, d, e). The center panel shows the distance from the target structure to the final structure with a gradient from white being the closest and the red being the farthest. The right panel shows the magnified region where the mutation is present. Note, in particular, the significant distortions introduced in lateral seam strands in (d) and (e).

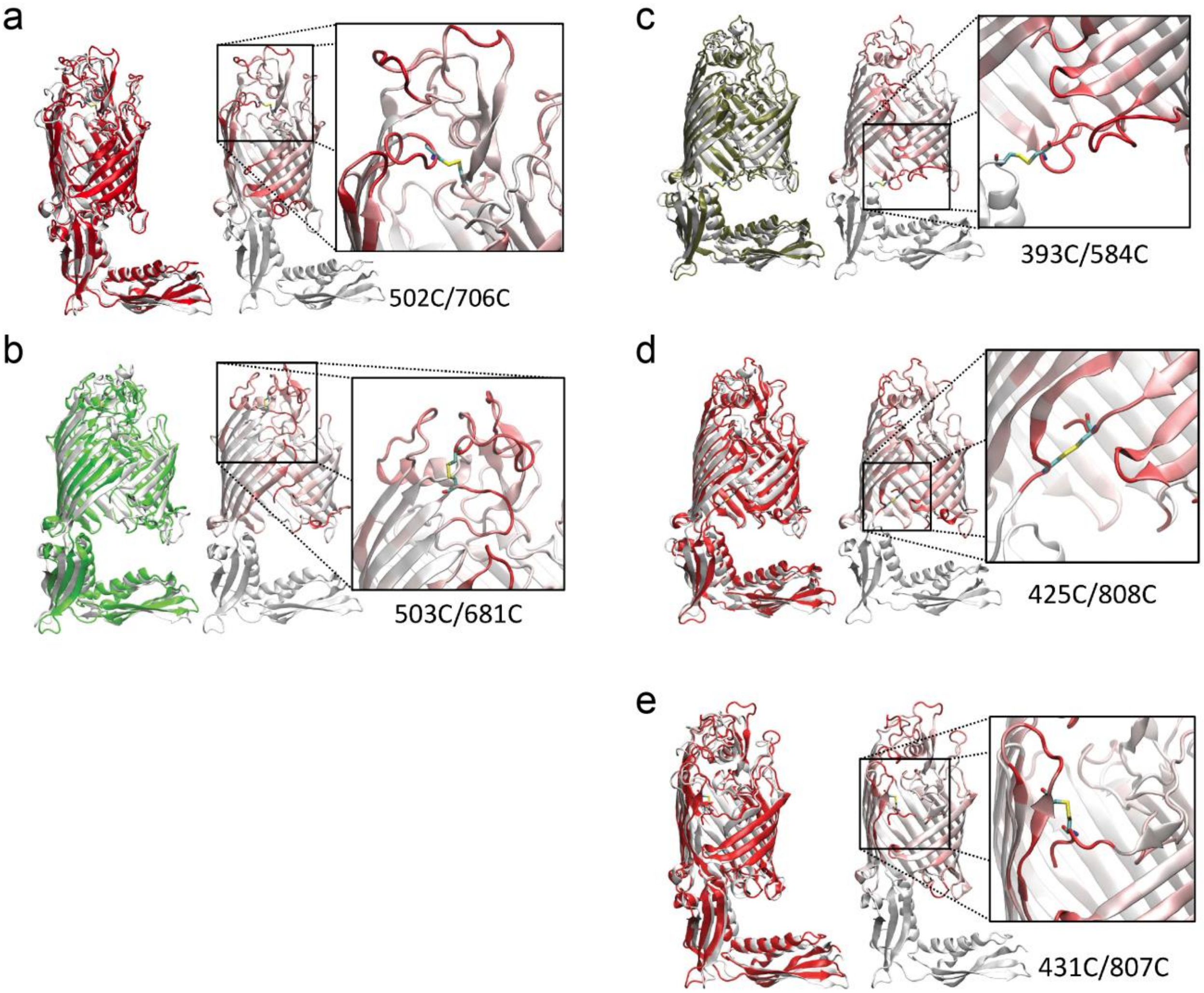

Supplementary Figure 13. EM analysis of the BAM/MBP-EspP complex in E3 nanodiscs. **a**. A representative motion corrected micrograph. The scale bar represents 100 nm. **b**. Selected 2D classes from 2D classification. **c**. Eight 3D classes from 3D classification. **d**. Refined 3D electron density map to 7.0 Å resolution. Plots for FSC resolution estimation (**e**), angular distribution for all particles (**f**), and local resolution (**g**) are shown for the final reconstruction.

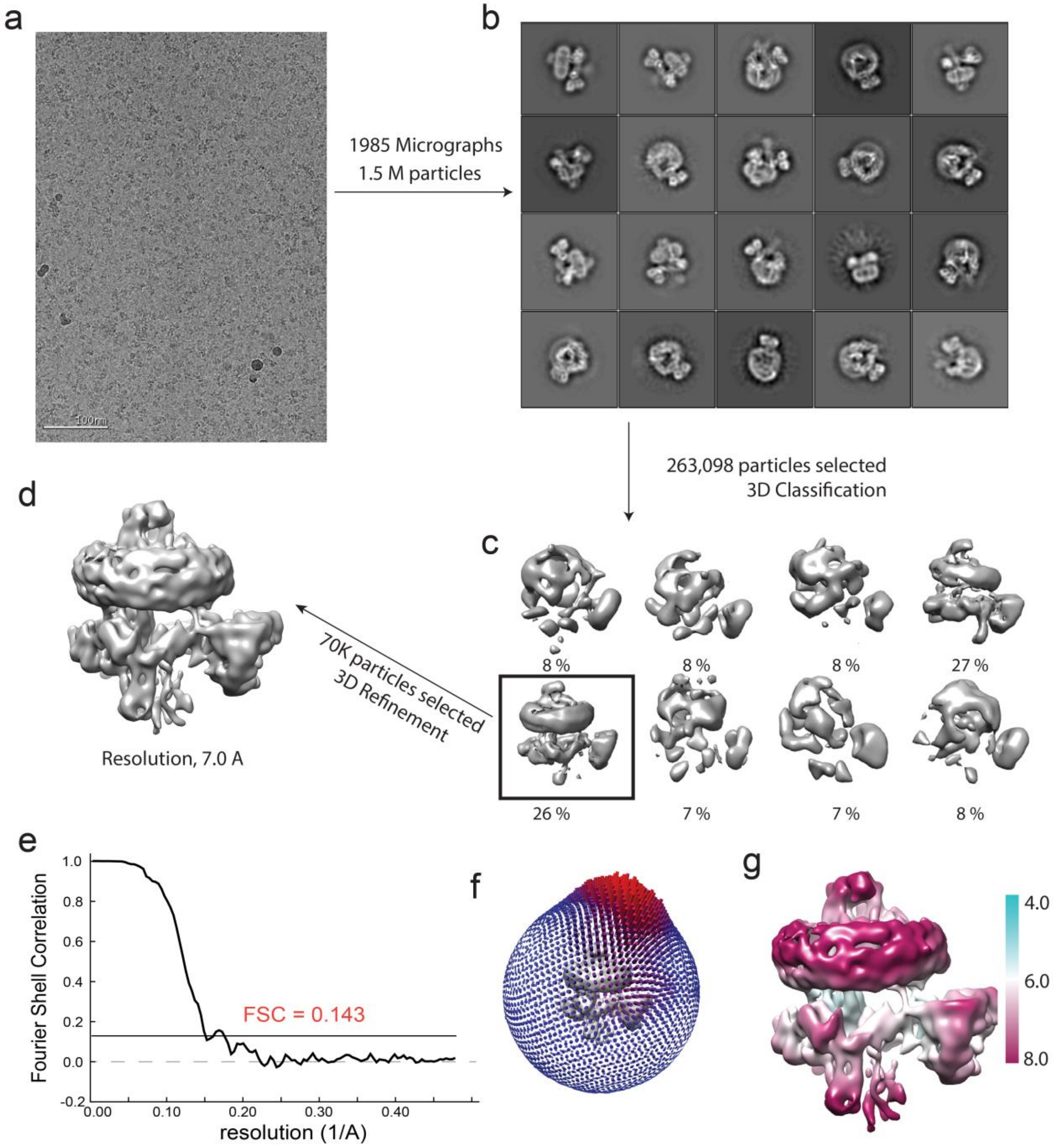

Supplementary Figure 14. **CryoEM reconstruction of the BAM/EspP$^{\beta 9-12}$ complex in detergent. a**. A representative motion corrected micrograph. The scale bar represents 200 nm. **b**. Selected 2D classes from 2D classification. **c**. Six 3D classes from 3D classification. **d**. Refined 3D electron density map to 3.4 Å resolution. **e**. Plot for FSC resolution estimation for the final 3D reconstruction.

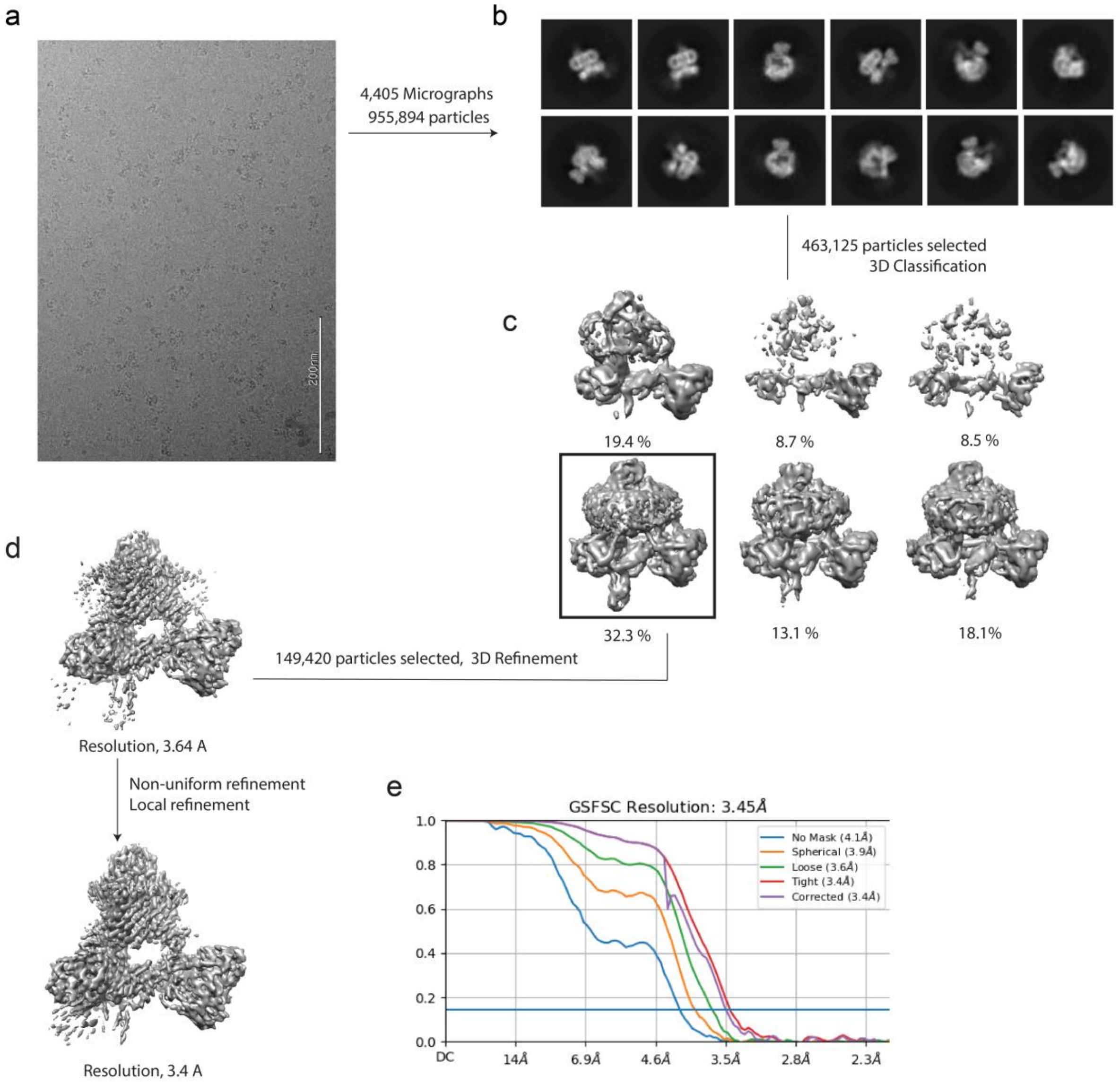

Supplementary Figure 15. Comparison of the BAM cryoEM structures in saposin and MSP1E3D1 nanodiscs. a. Structural alignment of the BAM-saposin structure (4.2 Å) with the inward-open structure (gray) (PDB ID 5D0O; RMSD 5.4 Å). **b**. Structural alignment of the BAM-saposin structure with our BAM-MSP1E3D1 structure (4.1 Å) (gray) (RMSD 1.6 Å). **c**. Structural alignment of BamA barrel and POTRA5 only from the BAM-saposin structure with that of the inward-open structure (gray). **d**. Structural alignment of BamA barrel and POTRA5 only from the BAM-saposin structure with our BAM-MSP1E3D1 structure (gray). The only significant change is along β1-4 which is apparently pushed inward to close the top of the barrel. **e**. An alignment of the cryoEM maps along the nanodisc regions from the BAM-saposin structure (magenta) and from our BAM-MSP1E3D1 structure (green). For reference, the location of BAM is indicated in gray cartoon; portions of the periplasmic domains have been removed. This image demonstrates that the density from the nanodisc appears thicker when using saposin compared to MSP1E3D1. Whether this observation can be attributed to the nanodisc proteins themselves, the difference in lipids used (total lipids vs polar lipids), or from the masking procedures used for the saposin reconstruction is unknown. **f**. Saposin reconstruction contoured at Level 0.012 vs the MSP1E3D1 reconstruction contoured at Level 0.02; and both contoured at Level 0.016 (**g**).

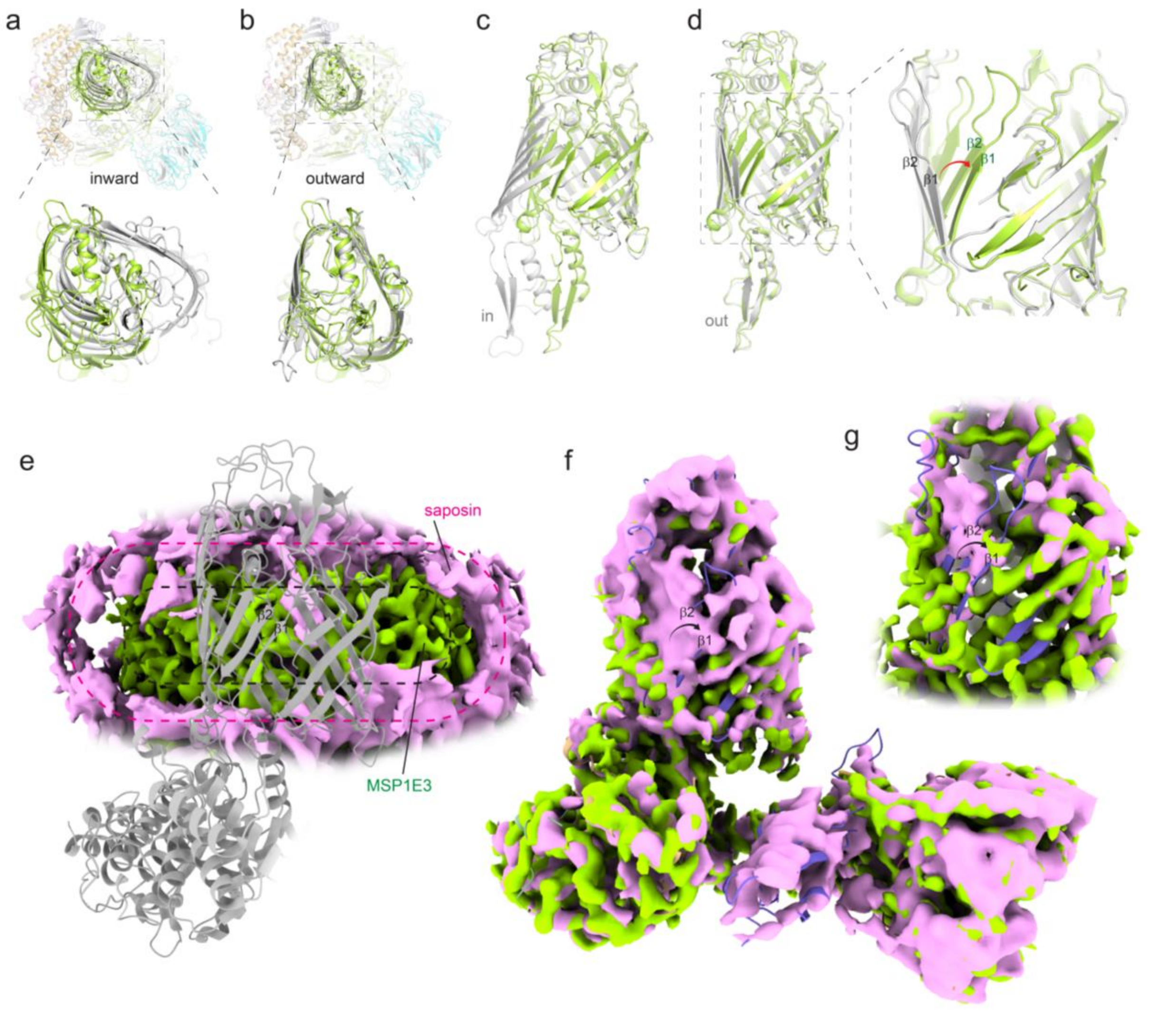

Supplementary Figure 16. Crystal contacts stabilize structures of the BAMΔB complex in the outward-open state. (left) For 5EKQ, the unit cell is shown, while for 5D0Q, two asymmetric copies in the unit cell are shown. **(right)** For each structure, a close-up of the POTRA domains, colored (1) grey, (2) cyan, (3) light blue, (4) dark blue, and (5) violet, is shown. The rest of BamA and BamC-E in that copy are colored purple. A neighboring BamA is shown in red (5EKQ) or green (5D0Q).

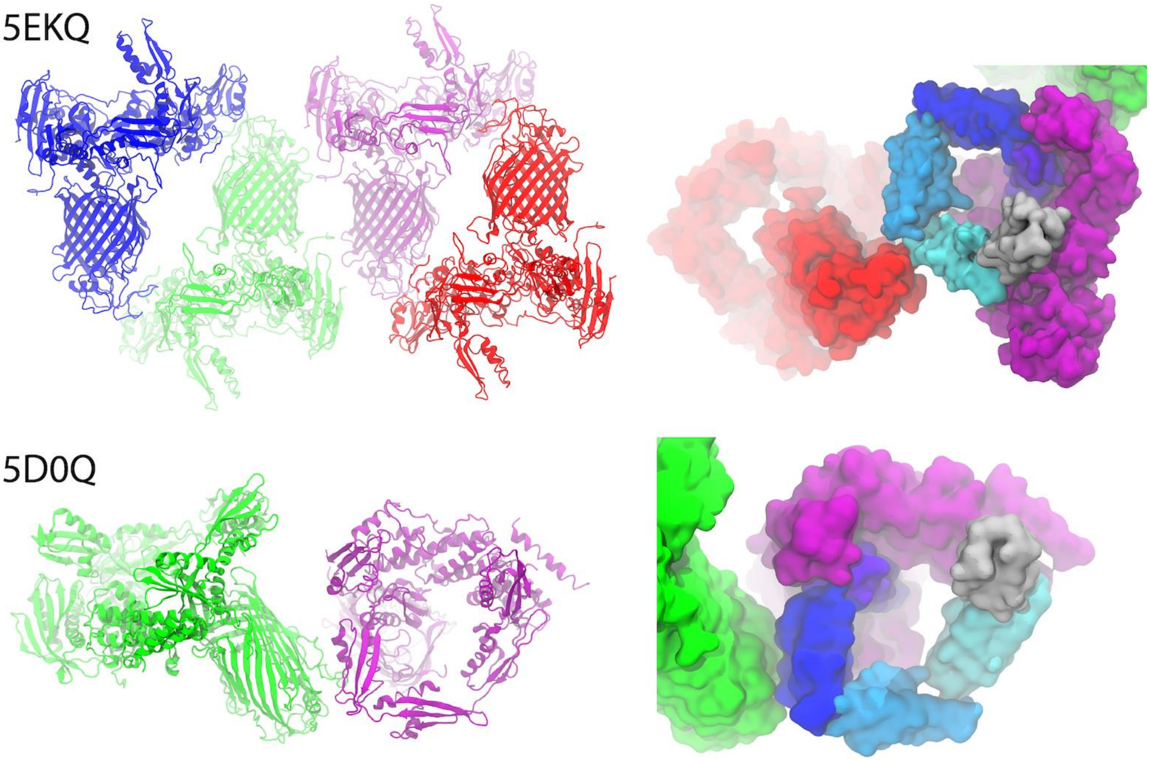

Supplementary Figure 17. Hybrid-barrel mechanism for OMP biogenesis by BAM. Looking down the top of barrel of BamA and the hybrid-barrel intermediate, an updated model for how BAM functions in OMP biogenesis is shown, considering our BAM/MBP-EspP structure (early intermediate) and the recently reported BAM/$BamA_{sub}$ structure (late intermediate; PDB ID 6V05). The top row is based on direct structural evidence, while the bottom row is based on hypothesis. The orange arrows indicate conformational switches within the barrel domain of BamA.

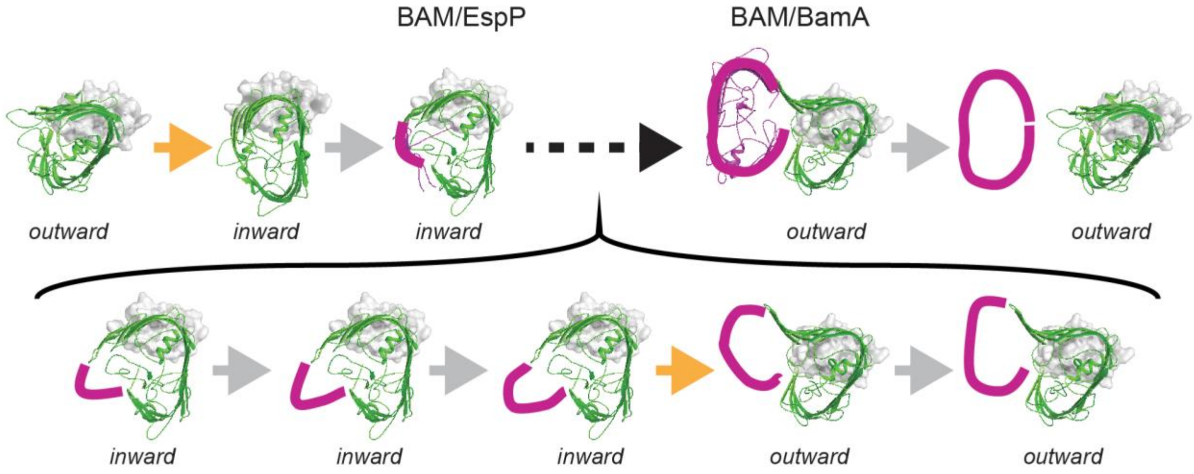

Supplementary Table 1. CryoEM data collection and model statistics. Summary of the data collection and refinement statistics for all cryoEM structures reported in this study.

	BAM_D1	BAM_E3	BAM_N2	BAM/EspP	BAM_Native	BAM_E3HR	BAM/EspP$^{\beta 9\text{-}12}$
**Data Collection**							
Microscope	Titan Krios	Titan Krios	Titan Krios	Titan Krios	Titan Krios	Titan Krios	Titan Krios
Magnification	81,000	81,000	81,000	81,000	81,000	81,000	×81,000
Voltage (kV)	300	300	300	300	300	300	300
Detector	K2	K2	K2	K3	K3	K3	K3
Software	Leginon	Leginon	Leginon	Leginon	Leginon	Leginon	Leginon
Exposure (s/fr)	0.2	0.2	0.2	0.078	0.065	0.065	0.065
# of frames/mic	60	60	60	40	40	40	40
Total dose (e-/Å^2)	43.85	43.85	43.85	51.14	44.9	44.9	44.77
Defocus (μm)	-1.5 to -2.5	-1.5 to -2.5	-1.5 to -2.5	-1.5 to -2.5	-1.5 to -2.5	-1.5 to -2.5	-1.0 to -2.0
Pixel size (Å)	0.69	0.69	0.69	0.525	0.525	0.525	0.54
**Image processing**							
Micrographs	961	1115	1111	1985	2247	5217	4,405
# Initial particles	783,635	813,270	1,196,641	1,529,450	256,440	8,053,211	955,894
# Final particles	39,504	73,038	72,244	70,031	54,989	1,121,059	149,420
Symmetry	C1	C1	C1	C1	C1	C1	C1
FSC threshold	0.143	0.143	0.143	0.143	0.143	0.143	0.143
Final resol. (Å)	8.0	6.9	7.5	7.0	5.9	4.0	3.4
**Model composition**							
# Residues	1,510	1,510	1,510	1614	1,510	1,510	1,578
**Model Refinement**							
Refinement	Phenix	Phenix	Phenix	Phenix	Phenix	Phenix	Phenix
R.m.s. deviations from ideal values							
Bond lengths (Å)	0.007	0.005	0.007	0.008	0.009	0.007	0.003
Bond angles (°)	0.988	0.875	1.039	1.262	1.438	1.038	0.674
**Validation**							
Ramachandran (%)							
Favored (%)	81.92	83.25	80.13	79.06	79.34	86.75	89.64
Allowed (%)	17.75	16.56	19.47	20.82	20.33	13.05	10.29
Disallowed (%)	0.33	0.2	0.4	0.12	0.33	0.20	0.07
Rotamer outliers (%)	1.32	0.82	1.32	0.23	0.0	0.08	10.0
Clashscore	64.43	50.14	66.48	59.09	75.43	21.42	10.74
Map CC (main)	0.86	0.80	0.78	0.84	0.77	0.74	0.83
Map CC (side)	0.84	0.78	0.77	0.83	0.75	0.73	0.82
**EMDB ID**	24476	24478	24477	24481	24475	24474	24473
**PDB ID**	7RI7	7RI9	7RI8	7RJ5	7RI6	7RI5	7RI4

Supplementary Table 2. Summary of primers and plasmids.

**Primer Name**	**Sequence**
Mal_F	TTTTCCATGGGGGCCAAAATCGAAGAAGG
Mal_R	AAAAGGATCCAGTCTGCGCGTCTTTCAGGGC
EspP(948-1300)_F	TTTTGGATCCATTGAACTGGTAAGCGCGCC
EspP(948-1300)_R	AAAACTCGAGTCAGAACGAGTAACGG
C690S/C700S_F	GATTACGAATCTGCGACTCAGGACGGCGCGAAAGACCTGTCT
C690S/C700S_R	CGATTTAGACAGGTCTTTCGCGCCGTCCTGAGTCGCAGATTC
N805C_F	GTTCCAGTTTTGCATCGGTAAAACC
N805C_R	GGTTTTACCGATGCAAAACTGGAAC
I806C_F	CAGTTTAACTGCGGTAAAACC
I806C_R	GGTTTTACCGCAGTTAAACTG
G807C_F	GTTTAACATCTGTAAAACCTGG
G807C_R	CCAGGTTTTACAGATGTTAAAC
G1226C_F	GCCCGTGCCTGTCTGGGCTAC
G1226C_R	GTAGCCCAGACAGGCACGGGC
G1228C_F	GCCGGTCTGTGCTACCAGTTC
G1228C_R	GAACTGGTAGCACAGACCGGC
Q1230C_F	CTGGGCTACTGTTTCGACCTG
Q1230C_R	CAGGTCGAAACAGTAGCCCAG
D1232C_F	GGCTACCAGTTCTGCCTGCTGGC
D1232C_R	GCCAGCAGGCAGAACTGGTAGCC
L1234C_F	GTTCGACCTGTGTGCTAACGGC
L1234C_R	GCCGTTAGCACACAGGTCGAAC
EspP_ β9-12_F	TTTGGTCTCGAGGTCAAAGTGACAGCCCGTGC
EspP_ β9-12_R	AAAGGTCTCGACCTCGTTCAGAAACGCTTTATAG
**Plasmids**	**Description/References**
pJH114xB	pJH114[1] with an extra copy of BamB added
pMSP1D1	Addgene; Gift from Stephen Sligar
pMSP1E3D1	Addgene; Gift from Stephen Sligar
pMSP2N2	Kindly provided by J. Psonis
pHis2/MBP−EspP	pHIS-Parallel2 vector; expression of MBP-EspP inclusion bodies
pCDF-1b	Novagen plasmid
pBAD/HisA	ThermoFisher plasmid
pRW1	pCDF-1b with promoter changed to araBAD
pRW1/MBP−EspP	pRW1 expressing MBP-EspP
pRW1/MBP−EspPβ9-12	pRW1 expressing MBP-EspPβ9-12
pRW1/MBP−EspPG1226C_β9-12	pRW1 expressing MBP-EspPG1226C_β9-12
pRW1/MBP−EspPG1228C_β9-12	pRW1 expressing MBP-EspPG1228C_β9-12
pRW1/MBP−EspPQ1230C_β9-12	pRW1 expressing MBP-EspPQ1230C_β9-12
pRW1/MBP−EspPD1232C_β9-12	pRW1 expressing MBP-EspPD1232C_β9-12
pRW1/MBP−EspPL1232C_β9-12	pRW1 expressing MBP-EspPL1232C_β9-12
pJH114C690S/C700S	pJH114 producing cysteine-less BamA
pJH114C690S/C700S/G807C	pJH114 producing cysteine-less BamA and G807C mutation
pJH114C690S/C700S/I806C	pJH114 producing cysteine-less BamA and I806C mutation

Supplementary References

1 Roman-Hernandez, G., Peterson, J. H. & Bernstein, H. D. Reconstitution of bacterial autotransporter assembly using purified components. *Elife* **3**, e04234, doi:10.7554/eLife.04234 (2014).
