# Supplementary material for: Plasticity within the barrel domain of BamA mediates a hybrid-barrel mechanism by BAM
Source: Nat Commun. 2021 Dec 8;12:7131. doi: 10.1038/s41467-021-27449-4 (PMC8655018; doi:10.1038/s41467-021-27449-4)
Supplement: Supplementary file 2 — Reporting Summary [file 41467_2021_27449_MOESM2_ESM.pdf]

nature research

Corresponding author(s): Nicholas Noinaj

Last updated by author(s): 11_02_2021

Reporting Summary

Nature Research wishes to improve the reproducibility of the work that we publish. This form provides structure for consistency and transparency in reporting. For further information on Nature Research policies, see our Editorial Policies and the Editorial Policy Checklist.

Statistics

For all statistical analyses, confirm that the following items are present in the figure legend, table legend, main text, or Methods section.

n/a	Confirmed	
☐	☒	The exact sample size (*n*) for each experimental group/condition, given as a discrete number and unit of measurement
☐	☒	A statement on whether measurements were taken from distinct samples or whether the same sample was measured repeatedly
☒	☐	The statistical test(s) used AND whether they are one- or two-sided<br>*Only common tests should be described solely by name; describe more complex techniques in the Methods section.*
☒	☐	A description of all covariates tested
☒	☐	A description of any assumptions or corrections, such as tests of normality and adjustment for multiple comparisons
☒	☐	A full description of the statistical parameters including central tendency (e.g. means) or other basic estimates (e.g. regression coefficient) AND variation (e.g. standard deviation) or associated estimates of uncertainty (e.g. confidence intervals)
☒	☐	For null hypothesis testing, the test statistic (e.g. F, t, r) with confidence intervals, effect sizes, degrees of freedom and P value noted<br>*Give P values as exact values whenever suitable.*
☒	☐	For Bayesian analysis, information on the choice of priors and Markov chain Monte Carlo settings
☒	☐	For hierarchical and complex designs, identification of the appropriate level for tests and full reporting of outcomes
☒	☐	Estimates of effect sizes (e.g. Cohen's d, Pearson's r), indicating how they were calculated

Our web collection on statistics for biologists contains articles on many of the points above.

Software and code

Policy information about availability of computer code

Data collection	Leginon, Digital Micrograph
Data analysis	Relion (v 3), cryoSPARC (v, PyMOL (v 2.3.2), COOT (v 0.9.4), ChimeraX (v 1.3); script for nanodiscs simulations (https://gist.github.com/wjiang/fef38d342ca51b7e89d282c653ddd1fa)

For manuscripts utilizing custom algorithms or software that are central to the research but not yet described in published literature, software must be made available to editors and reviewers. We strongly encourage code deposition in a community repository (e.g. GitHub). See the Nature Research guidelines for submitting code & software for further information.

Data

Policy information about availability of data

All manuscripts must include a data availability statement. This statement should provide the following information, where applicable:

- Accession codes, unique identifiers, or web links for publicly available datasets
- A list of figures that have associated raw data
- A description of any restrictions on data availability

CryoEM maps and models have been deposited into the Electron Microscopy Data Bank (EMDB) and Protein Data Bank (PDB) with the following accession numbers: BAM in D1 nanodiscs (EMD-24476 and PDB ID 7RI7), BAM in N2 nanodiscs (EMD-24477 and PDB ID 7RI8), low-resolution BAM in E3 nanodiscs (EMD-24478 and PDB ID 7RI9), high-resolution BAM in E3 nanodiscs (EMD-24474 and PDB ID 7RI5), BAM in nanodiscs prepared from E. coli outer membranes (EMD-24475 and PDB ID 7RI6), low-resolution BAM/EspP (EMD- 24481 and PDB ID 7RJ5), and BAM/EspP 9-12 (EMD-24473 and PDB ID 7RI4). Plasmids and other non-commercially available reagents used in this study are available from N.N. under a material transfer agreement with Purdue University.

Field-specific reporting

Please select the one below that is the best fit for your research. If you are not sure, read the appropriate sections before making your selection.

☒ Life sciences ☐ Behavioural & social sciences ☐ Ecological, evolutionary & environmental sciences

For a reference copy of the document with all sections, see nature.com/documents/nr-reporting-summary-flat.pdf

Life sciences study design

All studies must disclose on these points even when the disclosure is negative.

Sample size	For EM, a suitable number of samples were determined based on the resulting resolution desired; this has been summarized in the cryoEM data collection table. For the Western blots, sample sizes were usually dependent on repeating at least 2 times to confirm reproducibility.
Data exclusions	For cryoEM, particles were excluded based on the lack of a defined shape or secondary structure, indicating a mixed class not suitable for further inclusion in the data processing. For the crosslinking studies, those crosslinks not producing a notable band via SDS-PAGE/Western blot analysis was also excluded.
Replication	as indicated in the manuscript where applicable, experiments were performed in triplicate
Randomization	This study does not include randomization of a population, however, non-bias initial ab initio models were produced from an initially randomized model and then refined during the ab initial modeling process.
Blinding	this study does not necessitate blinding controls.

Reporting for specific materials, systems and methods

We require information from authors about some types of materials, experimental systems and methods used in many studies. Here, indicate whether each material, system or method listed is relevant to your study. If you are not sure if a list item applies to your research, read the appropriate section before selecting a response.

Materials & experimental systems

n/a	Involved in the study	
☐	☒	Antibodies
☒	☐	Eukaryotic cell lines
☒	☐	Palaeontology and archaeology
☐	☒	Animals and other organisms
☒	☐	Human research participants
☒	☐	Clinical data
☒	☐	Dual use research of concern

Methods

n/a	Involved in the study	
☒	☐	ChIP-seq
☒	☐	Flow cytometry
☒	☐	MRI-based neuroimaging

Antibodies

Antibodies used	anti-MBP (NEB; E8032S); anti-BamA (custom made, Precision Antibody); anti-DegP (gift); anti-SurA (gift); anti-HIS (Sigma; A7058)
Validation	NEB and Sigma provide validation of their antibodies on their website through the use of showing direct validation via immunoblotting of a sample and physical properties; no other statements were provide by the companies. Precision Antibody provided confirmation of the anti-BamA antibody which we have confirmed in lab by analysis and comparison of +/- EcBamA lysates. anti-DegP and anti-Sur A were gifts from other labs as noted in the manuscript and have been used for previous publications, including some of our own (Noinaj et al. Nature, 2013; Noinaj et al, Structure 2014).

Animals and other organisms

Policy information about studies involving animals; ARRIVE guidelines recommended for reporting animal research

Laboratory animals	n/a
Wild animals	n/a
Field-collected samples	n/a
Ethics oversight	only bacteria used for protein production was used in this study. That included BL21(DE3) cells which are routine cell strains for recombinant protein technoloy.

Note that full information on the approval of the study protocol must also be provided in the manuscript.
